# Establishment of Novel Prostate Cancer Risk Subtypes and A Twelve-Gene Prognostic Model

**DOI:** 10.3389/fmolb.2021.676138

**Published:** 2021-05-28

**Authors:** Enchong Zhang, Fujisawa Shiori, Mo Zhang, Peng Wang, Jieqian He, Yuntian Ge, Yongsheng Song, Liping Shan

**Affiliations:** ^1^ Department of Urology, Shengjing Hospital of China Medical University, Shenyang, China; ^2^ Department of Breast Endocrine Surgery, Tohoku University Hospital, Sendai, Japan

**Keywords:** prostate cancer, predictive model, prognosis, RhoBTB2, TNFRSF10C, systems biology

## Abstract

Prostate cancer (PCa) is the most common malignancy among men worldwide. However, its complex heterogeneity makes treatment challenging. In this study, we aimed to identify PCa subtypes and a gene signature associated with PCa prognosis. In particular, nine PCa-related pathways were evaluated in patients with PCa by a single-sample gene set enrichment analysis (ssGSEA) and an unsupervised clustering analysis (i.e., consensus clustering). We identified three subtypes with differences in prognosis (Risk_H, Risk_M, and Risk_L). Differences in the proliferation status, frequencies of known subtypes, tumor purity, immune cell composition, and genomic and transcriptomic profiles among the three subtypes were explored based on The Cancer Genome Atlas database. Our results clearly revealed that the Risk_H subtype was associated with the worst prognosis. By a weighted correlation network analysis of genes related to the Risk_H subtype and least absolute shrinkage and selection operator, we developed a 12-gene risk-predicting model. We further validated its accuracy using three public datasets. Effective drugs for high-risk patients identified using the model were predicted. The novel PCa subtypes and prognostic model developed in this study may improve clinical decision-making.

## Introduction

Prostate cancer (PCa) is highly intractable and incurable after metastasis. It is the leading type of noncutaneous cancer in males globally (Lee et al., 2017; Wang et al., 2018). Conventional therapeutic strategies for PCa are insufficient owing to tumor heterogeneity and complex molecular mechanisms of metastasis, leading to wide variation in outcomes (Wang et al., 2018). The clinical management of PCa includes surgery, androgen-deprivation therapy, ablative therapies, chemotherapy, radiation therapy, and immune therapy (Evans, 2018). Despite progress in therapeutic strategies, the treatment efficacy for advanced PCa is still low (Vlachostergios et al., 2017). In the context of precision medicine, the classification of PCa according to molecular features and prognosis will undoubtedly unlock effective targeted treatment strategies.

The mechanism underlying PCa heterogeneity and metastasis is highly complex; even within the same tumor, distinct phenotypes and characteristics exist (Meacham and Morrison, 2013). Multiple genomic changes contribute to PCa progression at the early stage and could define molecular subtypes. In our previous study (Zhang et al., 2020), we identified four subtypes of PCa based on immune-related gene sets. Labrecque et al., 2019 defined four novel subtypes of metastatic castration-resistant prostate cancer based on a 26-gene signature as well as distinct features of androgen receptor responses and *NEUROI* and *NEUROII* gene expression levels (Labrecque et al., 2019). Multiple molecular mechanisms work together to influence the development, progression, and outcome of PCa and thus precise molecular characterization can improve the accuracy of clinical decision-making.

For patients with PCa, an elevated hypoxic status is related to a more aggressive and advanced disease; hypoxia reduction could increase immunity and the response to specific immunotherapies (Jayaprakash et al., 2018). Additionally, prostate is an androgen-dependent organ, and androgen interactions with androgen receptors play a key role in the progression of PCa. Endocrine therapy in PCa is aimed at lowering serum androgen levels and inhibiting androgen receptor; when this approach fails, PCa advances to a hormone-resistant state (Heinlein and Chang, 2004; Shafi et al., 2013). The PI3K-AKT-mTOR pathway interacts with multiple cellular cascades, further promoting PCa progression and aggression, and drugs targeting this pathway are employed in clinical settings (Shorning et al., 2020). E2F and MYC synergistically contribute to cell cycle regulation and are involved in tumorigenesis (Liu et al., 2015). Metabolic adaptation is pivotal for malignancy given the high energy demand, and glycolytic, fatty acid biosynthesis, and oxidative phosphorylation contribute to proliferation and worse outcomes in PCa (Schöpf et al., 2016; Xiao et al., 2018; Balusamy et al., 2020). Machine learning has become increasingly advantageous in cancer research in the era of big data, enhancing disease prediction and prognosis (Kourou et al., 2015; Qiu et al., 2020a; Qiu et al., 2020b). We classified samples into three subtypes with different patterns of pathway enrichment. We hypothesized that a multi-pathway approach could enable the subclassification of PCa with different phenotypes, functions, and clinical characteristics. Here, we exploited nine pivotal PCa-related pathways based on a literature review to divide PCa samples into three subtypes, Risk_H, Risk_M, and Risk_L, with high, middle, and low risks, respectively. Next, we explored the characteristics of subtypes with respect to the tumor microenvironment, proliferation, single nucleotide variation, and copy number variation. Then, we explored the factors contributing to the observed differences in prognosis. We constructed a risk-predicting model based on genes associated with the high-risk subtype to make the prognosis calculable. Finally, we validated the efficacy of the risk model in an internal and three external validation cohorts and predicted drugs with high sensitivity in patients with PCa classified as high risk.

## Materials and Methods

### The Cancer Genome Atlas Data Processing

RNA sequencing (RNA-seq) data (Workflow type: HTSeq-Counts), single nucleotide variants (SNV) (Workflow type: MuSE Variant Aggregation and Masking), copy number variants (CNV) (Data type: Masked Copy Number Segment), and clinical phenotypes for patients with PCa in TCGA were downloaded. RNA-seq data were normalized using the DESeq2 R package (Love et al., 2014). Tumor mutational burden (TMB) for each patient was determined from SNV data using the maftools R package. Patients with incomplete survival data or a follow-up duration of less than 30°days were excluded. In total, 484 patients were retained for the clustering analysis. The progression-free interval (PFI) was obtained from an integrated TCGA pan-cancer Clinical Data Resource (Liu et al., 2018). The clinical phenotypes of 484 patients are shown in Table 1. Survival outcomes are shown in Supplementary Table S1. The proliferation scores for patients in TCGA were obtained from Thorsson et al. (Thorsson et al., 2018). For the identification of a prognostic model, patients in TCGA were randomly divided into a training group and internal validation group using the caret R package (Kuhn, 2008). Furthermore, the AR activity scores and TMPRSS2−ERG fusion status of patients with PCa were obtained from cBioPortal (https://www.cbioportal.org/) and The Tumor Fusion Gene Data Portal (https://www.tumorfusions.org/) (Cerami et al., 2012; Gao et al., 2013; Yoshihara et al., 2015).

**TABLE 1:** The disease-related clinical information of patients with PCa included in the study.

Characteristics	Value
Patients (*n*)	484
Age (year), median (IQR)	62.0 (56.0–66.0)
PSA (ng/ml), median (IQR)	7.5 (5.1–11.3)
Pathological Gleason score, *n* (%)	
≤6	43 (9.0%)
7 (3+4)	143 (30.0%)
7 (4+3)	100 (21.0%)
8	56 (11.7%)
9–10	135 (28.3%)
Prior malignancy, *n* (%)	
No	450 (94.3%)
Yes	27 (5.7%)
Race, *n* (%)	
Asian	12 (2.5%)
Whit, American Indian or Alaska native	398 (83.4%)
Black or African American	55 (11.6%)
NA	12 (2.5%)
Residual tumor, *n* (%)	
R0	301 (63.1%)
R1	15 (3.1%)
R2	142 (29.8%)
Rx	5 (1.0%)
NA	14 (3.0%)
Clinical M, *n* (%)	
M0	437 (91.6%)
M1a or M1c	2 (0.4%)
NA	38 (8.0%)
Pathological T, *n* (%)	
T1c	2 (0.4%)
T2a	13 (2.7%)
T2b	10 (2.1%)
T2c	160 (33.5%)
T3a	151 (31.7%)
T3b	129 (27.0%)
T4	9 (1.9%)
NA	3 (0.7%)
Pathological N, *n* (%)	
N0	329 (69.0%)
N1	78 (16.4%)
NA	70 (14.6%)
Diagnostic CT or MRI, *n* (%)	
No evidence of extraprostatic extension	196 (41.1%)
Equivocal	6 (1.3%)
Extraprostatic extension localized	22 (4.6%)
Extraprostatic extension	9 (1.9%)
NA	244 (51.1%)

DFS, Disease-free survival; IQR, interquantile range; NA, not analyzed; PCa, prostate cancer; PSA, Prostate-specific antigen.

### Validation Data Set Processing

Data sets from Gene Expression Omnibus (GEO) and cBioPortal for Cancer Genomics were used to validate the accuracy of the prognostic model (Barrett et al., 2009; Cerami et al., 2012; Gao et al., 2013). GSE70769 was obtained using the GEOquery R package from the GEO database (Davis and Meltzer, 2007; Ross-Adams et al., 2015). The datasets DKF2018 and MSKCC2010 were downloaded from the cBioPortal database. Patients with incomplete survival data or a follow-up duration of less than 30°days were excluded.

### Single-Sample Gene Set Enrichment Analysis

Based on a literature review, nine gene sets associated with PCa were selected (Gann et al., 1994; Heinlein and Chang, 2004; Kaseb et al., 2007; Koh et al., 2010; Milosevic et al., 2012; Edlind and Hsieh, 2014; Ippolito et al., 2016; Xiao et al., 2018). HALLMARK_ANDROGEN_RESPONSE, HALLMARK_E2F_TARGETS, HALLMARK_FATTY_ACID_METABOLISM, 'HALLMARK_GLYCOLYSIS, HALLMARK_HYPOXIA, HALLMARK_MYC_TARGETS_V1, HALLMARK_MYC_TARGETS_V2, HALLMARK_OXIDATIVE_PHOSPHORYLATION, and HALLMARK_PI3K_AKT_MTOR_SIGNALING gene sets were downloaded from the Molecular Signatures Database v7.2 (Liberzon et al., 2015). ssGSEA based on these nine gene sets was performed using the GSVA R package (Hänzelmann et al., 2013). The parameter settings were as follows: method = “ssgsea,” kcdf = “Gaussian,” abs.ranking = TRUE. Patients from TCGA (*n* = 484) were evaluated using the ssGSEA algorithm and enrichment scores were obtained for each gene set.

### Consensus Clustering

Consensus clustering was performed with the ssGSEA scores for patients (TCGA, *n* = 484) using the ConsensusClusterPlus R package (Wilkerson and Hayes, 2010). The number of subsamples was 100, proportion of items per sample was 0.8, and proportion of features per sample was 1. Hierarchical clustering was used. The adjacency distance matrix was determined as (1-Pearson correlation coefficient). Default settings were used for other parameters.

### Principal Coordinate Analysis

RNA-seq data in Counts were normalized using the DESeq2 R package (Love et al., 2014) and used in a principal coordinate analysis (PCA). The normalized Counts matrix was transformed into a Bray–Curtis dissimilarity matrix using the vegan R package. Then, PCA was conducted using the ape R package. Owing to the large sample size, means and standard errors of principal coordinate values were used to display the separation among subtypes, as described previously (Wu et al., 2020). Finally, PERMANOVA with 10,000 permutations was performed to determine the statistical significance of the separation in PCA.

### Deconvolution Algorithms

CIBERSORTx was used to analyze the immune composition in the microenvironment of PCa tissues from TCGA (Steen et al., 2020) assuming two modules. RNA-seq data in TPM format were uploaded as the mixture file. Impute Cell Fractions and LM22 (22 immune cell types) were selected for the signature matrix file. Additionally, 100 permutations were performed for the statistical analysis. Other parameters were set according to Tutorial 2 on the CIBERSORTx website.

xCell is a web-tool for cell type enrichment analyses of gene expression data for 64 immune and stroma cell types (Aran et al., 2017). According to the recommended guidelines, RNA-seq data were input in TPM format into xCell and “xCell (*N* = 64)” was selected as the gene signature.

The ESTIMATE algorithm can estimate tumor purity by calculating the ratio of stromal to immune cells based on gene expression data (Yoshihara et al., 2013). The Estimate R package was used to analyze. normalized RNA-seq data by this algorithm.

### Weighted Correlation Network Analysis

A weighted correlation network analysis (WGCNA) can be used find phenotype-associated gene modules (Langfelder and Horvath, 2008; Li et al., 2019). RNA-seq data in TPM format were used as the input for a WGCNA. Twelve was set as the soft power threshold to construct a network that simultaneously satisfied a scale-free topology and high connectivity. Pearson correlation coefficients for the relationships between ssGSEA scores and gene modules were calculated. The correlations between the gene significance value and module membership of genes in a module were explored by a Pearson correlation analysis.

### Least Absolute Shrinkage and Selection Operator Regression

LASSO regression was performed on data for patients in training group using the glmnet R package (Fonti and Belitser, 2017). Genes most highly related to E2F and MYC ssGSEA scores were used as inputs. During the selection of genes, the C-index after 10-fold cross-validation indicated the effect of different screening strategies. Genes with the maximal C-index values were included in the prognostic model with the following parameter settings: family = Cox, type.measure = C, parallel = TRUE, with default settings for other parameters.

### Time-Dependent Receiver Operating Characteristic Curve Analysis

The accuracy of the prognostic model was determined by a tdROC analysis using the survivalROC R package. The endpoints were follow-up times of 1, 3, and 5°years. The area under the curve in the tdROC analysis was used to quantify accuracy. AUC values were obtained for the training group, internal validation group, and three external validation groups (GSE70769, DKF 2018, and MSKCC 2010).

### Survival Analysis

The log-rank test and Cox regression for survival analyses were completed using the survival R package. The survival curve was plotted using the survminer R package. DFS, PFI, and biochemical recurrence-free survival were used as clinical outcomes. Follow-up time was evaluated in units of years. Finally, univariate and multivariate Cox regression analyses were used to explore whether the prognostic model is an independent predictor of prognosis.

### Drug Target Prediction

Based on CTRP version 2 and PRISM databases, drug sensitivities of high-risk patients identified using the model were predicted by ridge regression based on gene expression levels. The analysis was implemented in the pRRophetic R package (Geeleher et al., 2014). Components with significantly lower areas under the dose–response curve (dr-AUC) in high-risk patients were selected first. Next, Spearman’s correlation coefficients for the relationship between the dr-AUC and risk score were obtained. Components with significantly negative rho (less than −0.3) were retained.

### Statistical Analysis

All statistical analyses were completed in R version 3.6.3. Chi-squared tests and Fischer’s exact tests were used to compare discrete variables between or among groups. Continuous variables were compared using the Wilcoxon test (two groups) and the Kruskal–Wallis test (three or more groups). *p* < 0.05 was considered significant.

## Results

### Identification of Three Subtypes With Different Risk Levels

**FIGURE 1:**
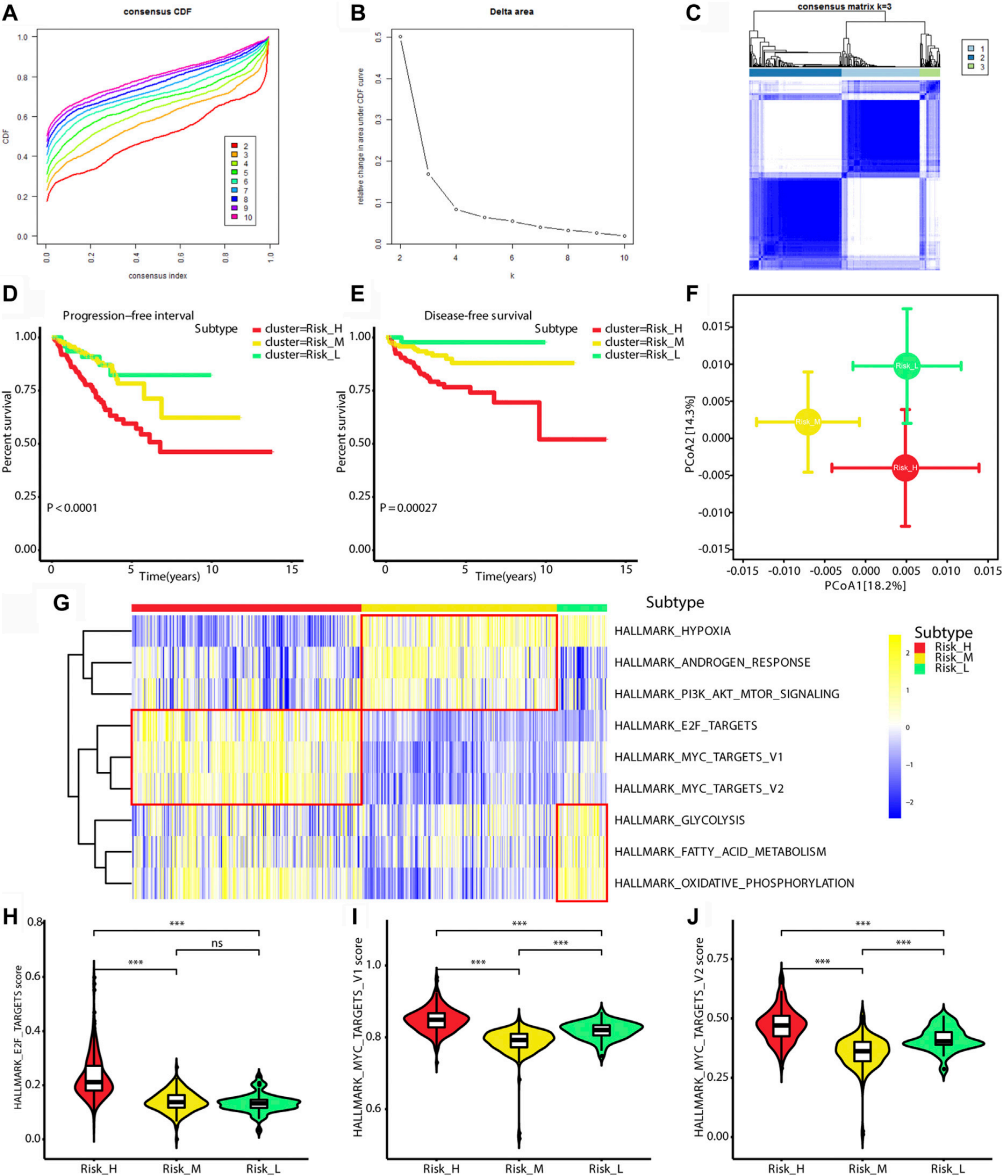
Identification of three subtypes with different prognosis. **(A)** The CDF curve under different values of k. The value of k represents the number of clusters during the consensus cluster. When the optimal k value is reached, the area under the CDF curve will not significantly increase with the increase of k value. **(B)** Relative change in area under CDF curve under different values of k. **(C)** The consensus matrix obtained when k = 3. Consistency values range from 0 to 1, 0 means never clustering together (white), 1 means always clustering together (dark blue). **(D)** Survival curves for progression-free interval of patients in the different subtypes. **(E)** Survival curves for disease-free survival of patients in the different subtypes. **(F)** PCA of Bray-Curtis dissimilarities obtained for the transcriptional profiles. The circles and error bars indicate the mean and standard errors of the mean. PERMANOVA test with 10,000 permutations were performed to calculate *p* value. **(G)** The heatmap shows ssGSEA scores levels among three subtypes. **(H)** The violin plot shows ssGSEA score of HALLMARK_E2F_TARGETS is significantly highest in Risk_H subtype. **(I)** The violin plot shows ssGSEA score of HALLMARK_MYC_TARGETS_V1 is significantly highest in Risk_H subtype. **(J)** The violin plot shows ssGSEA score of HALLMARK_MYC_TARGETS_V2 is significantly highest in Risk_H subtype. (PCa, prostate cancer; CDF, cumulative distribution function; PCA, principal coordinate analysis. * means *p* < 0.05, ** means *p* < 0.01, *** means *p* < 0.001, ns means *p* > 0.05, and *p* < 0.05 is defined as statistically significant).

Based on ssGSEA scores for nine PCa-associated gene sets, a consensus clustering analysis was performed for subtype identification. The cumulative distribution function (CDF) and relative change in the area under the CDF curve are shown in Figures 1A,B, respectively. According to Monti et al. (Qiu et al., 2020b), the optimal k-value is determined by a number of factors; one criterion is that when the optimal k-value is reached, the area under the CDF curve will not increase significantly with increases in k. We first set k = 4, indicating that the cohort could be divided into up to four subtypes. However, one cluster consisted of only a single patient when k = 4. Additionally, the cluster-consensus value for each cluster was not large enough under k = 4 (Supplementary Figure S1). Therefore, we focused on k = 3, dividing patients into three clusters (Figure 1C). In particular, according to prognostic features shown in Figures 1D,E, the clusters were defined as a high-risk subtype (Risk_H), moderate-risk subtype (Risk_M), and low-risk subtype (Risk_L). In a PCA, there was significant separation among the three subtypes (Figure 1F, PERMANOVA *p* < 0.05). Since these subtypes were identified based on ssGSEA scores, the levels of nine PCa-associated gene sets in the three subtypes were displayed in a heatmap in Figure 1G. We found that HALLMARK_HYPOXIA, HALLMARK_ANDROGEN_RESPONSE, and HALLMARK_PI3K_AKT_MTOR_SIGNALING were enriched in Risk_M. HALLMARK_E2F_TARGETS, HALLMARK_MYC_TARGETS_V1, and HALLMARK_MYC_TARGETS_V2 were enriched in Risk_H. HALLMARK_GLYCOLYSIS, HALLMARK_FATTY_ACID_METABOLISM, and HALLMARK_OXIDATIVE_PHOSPHORYLATION were enriched in Risk_L. As shown in Figures 1H–J, the ssGSEA scores for HALLMARK_E2F_TARGETS, HALLMARK_MYC_TARGETS_V1, and HALLMARK_MYC_TARGETS_V2 were significantly higher in Risk_H than in Risk_L and Risk_M (Wilcoxon test *p* < 0.001). Furthermore, the PSA, Gleason score, residual tumor, pathological T, and survival outcome were associated with the subtype status (Table 2). Collectively, these data indicated that PCa could be divided into three subtypes based on the degree of enrichment of factors related to the androgen response, hypoxia, PI3K/AKT/MTOR signaling, E2F activity, MYC activity, glycolysis, fatty acid metabolism, and oxidative phosphorylation. The Risk_H subtype, with enrichment for E2F and MYC activity, showed a worse prognosis.

**TABLE 2:** The association between subtypes and clinicopathologic variables of prostate cancer.

clinicopathologic variables	Subtype			*P*
	Risk_L (*n* = 51)	Risk_M (*n* = 199)	Risk_H (*n* = 234)	
Age (year), median (IQR)	61.0 (56.0–67.5)	61.0 (56.0–66.0)	62.0 (57.0–66.0)	0.307^a^
PSA (ng/ml), median (IQR)	6.1 (4.2–10.0)	7.2 (5.0–10.8)	7.8 (5.2–12.8)	0.017^a^
Pathological Gleason score, *n* (%)				< 0.001^b^
≤6	8 (15.7%)	16 (8.0%)	19 (8.1%)	
7 (3+4)	18 (35.3%)	75 (37.7%)	49 (20.9%)	
7 (4+3)	10 (19.6%)	44 (22.1%)	47 (20.1%)	
8	3 (5.9%)	28 (14.1%)	30 (12.8%)	
9–10	12 (23.5%)	36 (18.1%)	89 (38.0%)	
Prior malignancy, *n* (%)				0.562^c^
No	50 (98.0%)	187 (94.0%)	219 (93.6%)	
Yes	1 (2.0%)	12 (6.0%)	15 (6.4%)	
Race, *n* (%)				0.804^c^
Asian	1 (2.0%)	3 (1.5%)	8 (3.4%)	
Whit, American Indian or Alaska native	43 (84.3%)	169 (84.9%)	192 (82.1%)	
Black or African American	5 (9.9%)	22 (11.1%)	27 (11.5%)	
NA	2 (3.8%)	5 (2.5%)	7 (3.0%)	
Residual tumor, *n* (%)				
R0	31 (60.8%)	138 (69.3%)	139 (59.4%)	0.035^b^
Rx/R1/R2	19 (37.3%)	53 (26.6%)	91 (38.9%)	
NA	1 (1.9%)	8 (4.1%)	4 (1.7%)	
Clinical M, *n* (%)				1.000^c^
M0	49 (96.1%)	182 (91.5%)	213 (91.0%)	
M1a or M1c	0 (0.0%)	1 (0.5%)	1 (0.4%)	
NA	2 (3.9%)	16 (8.0%)	20 (8.6%)	
Pathological T, *n* (%)				0.027^c^
T1c	0 (0.0%)	2 (1.0%)	0 (0.0%)	
T2a	2 (3.9%)	4 (2.0%)	7 (3.0%)	
T2b	2 (3.9%)	3 (1.5%)	5 (2.1%)	
T2c	18 (35.3%)	84 (42.2%)	59 (25.2%)	
T3a	16 (31.4%)	60 (30.2%)	81 (34.6%)	
T3b	12 (23.5%)	63 (21.6%)	75 (32.1%)	
T4	1 (2.0%)	2 (1.0%)	6 (2.6%)	
NA	0 (0.0%)	1 (0.5%)	1 (0.4%)	
Pathological N, *n* (%)				0.141^b^
N0	31 (60.8%)	149 (74.9%)	155 (66.2%)	
N1	7 (13.7%)	26 (13.1%)	46 (19.7%)	
NA	13 (25.5%)	24 (12.0%)	33 (14.1%)	
Outcome, *n* (%)				
DFS	1 (2.0%)	15 (7.5%)	41 (17.5%)	<0.001^b^
Disease free	50 (98.0%)	184 (92.5%)	193 (82.5.0%)	

*p* values were calculated by the Kruskal test (a), Chi-square test (b) or Fisher’s exact test (c). DFS, Disease-free survival; IQR, interquartile range; NA, not analyzed; PCa, prostate cancer; PSA, Prostate-specific antigen.

### Risk_H Subtype is Associated With a Highly Proliferative State

**FIGURE 2:**
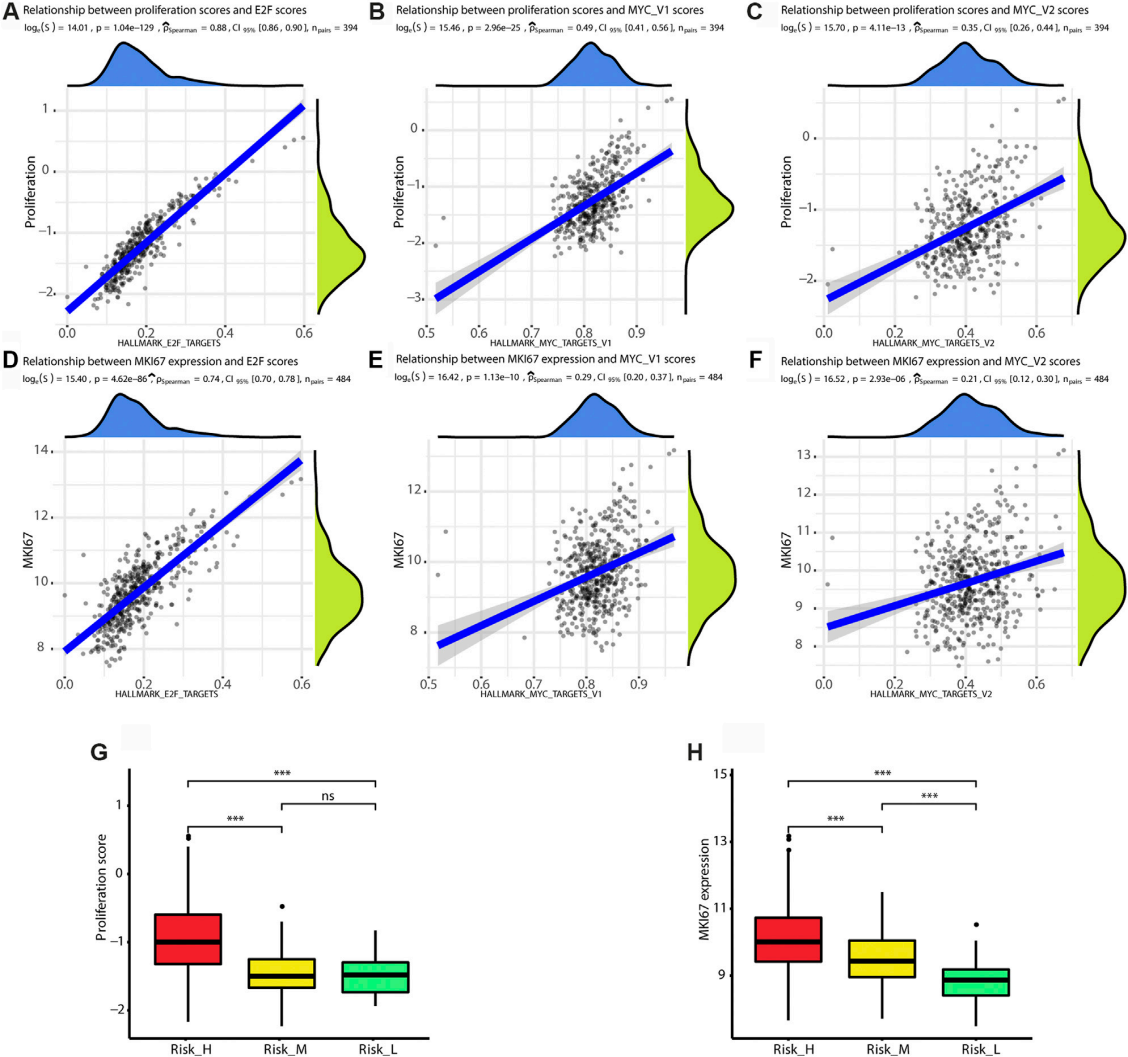
Risk_H subtype shows high proliferation status. **(A)** The scatter plot shows ssGSEA score of HALLMARK_E2F_TARGETS is positively correlated with proliferation score (Spearman rho = 0.88, *p* < 0.01). **(B)** The scatter plot shows ssGSEA score of HALLMARK_MYC_TARGETS_V1 is positively correlated with proliferation score (Spearman rho = 0.49, *p* < 0.01). **(C)** The scatter plot shows ssGSEA score of HALLMARK_MYC_TARGETS_V2 is positively correlated with proliferation score (Spearman rho = 0.35, *p* < 0.01). **(D)** The scatter plot shows ssGSEA score of HALLMARK_E2F_TARGETS is positively correlated with expression of *MKI67* (Spearman rho = 0.74, *p* < 0.01). **(E)** The scatter plot shows ssGSEA score of HALLMARK_MYC_TARGETS_V1 is positively correlated with expression of *MKI67* (Spearman rho = 0.29, *p* < 0.01). **(F)** The scatter plot shows ssGSEA score of HALLMARK_MYC_TARGETS_V2 is positively correlated with expression of *MKI67* (Spearman rho = 0.21, *p* < 0.01). **(G)** The boxplot shows the proliferation score is the highest in Risk_H subtype. **(H)** The boxplot shows the expression of *MKI67* is the highest in Risk_H subtype. (PCa, prostate cancer. * means *p* < 0.05, ** means *p* < 0.01, *** means *p* < 0.001, ns means *p* > 0.05, and *p* < 0.05 is defined as statistically significant).

We found that the ssGSEA scores for the E2F and MYC gene sets were positively correlated with proliferation scores (E2F score: rho = 0.88, *p* < 0.01; MYC_V1 score: rho = 0.49, *p* < 0.01; MYC_V2 score: rho = 0.35, *p* < 0.01), as shown in Figures 2A–C. Owing to the close relationship between KI67 expression and proliferation, the correlation between the expression of *MKI67* and ssGSEA scores was also explored. The levels of *MKI67* were positively correlated with ssGSEA scores for the E2F and MYC gene sets (E2F score: rho = 0.74, *p* < 0.01; MYC_V1 score: rho = 0.29, *p* < 0.01; MYC_V2 score: rho = 0.21, *p* < 0.01), as shown in Figures 2D–F. Finally, the proliferation scores and *MKI67* levels were highest in Risk_H among the three subtypes (Kruskal–Wallis test *p* < 0.001), as shown in Figures 2G,H. Taken together, tumors classified as Risk_H had higher levels of proliferation.

### Known Subtypes Associated With a Poor Prognosis Were Overrepresented in the Risk_H Subtype

**FIGURE 3:**
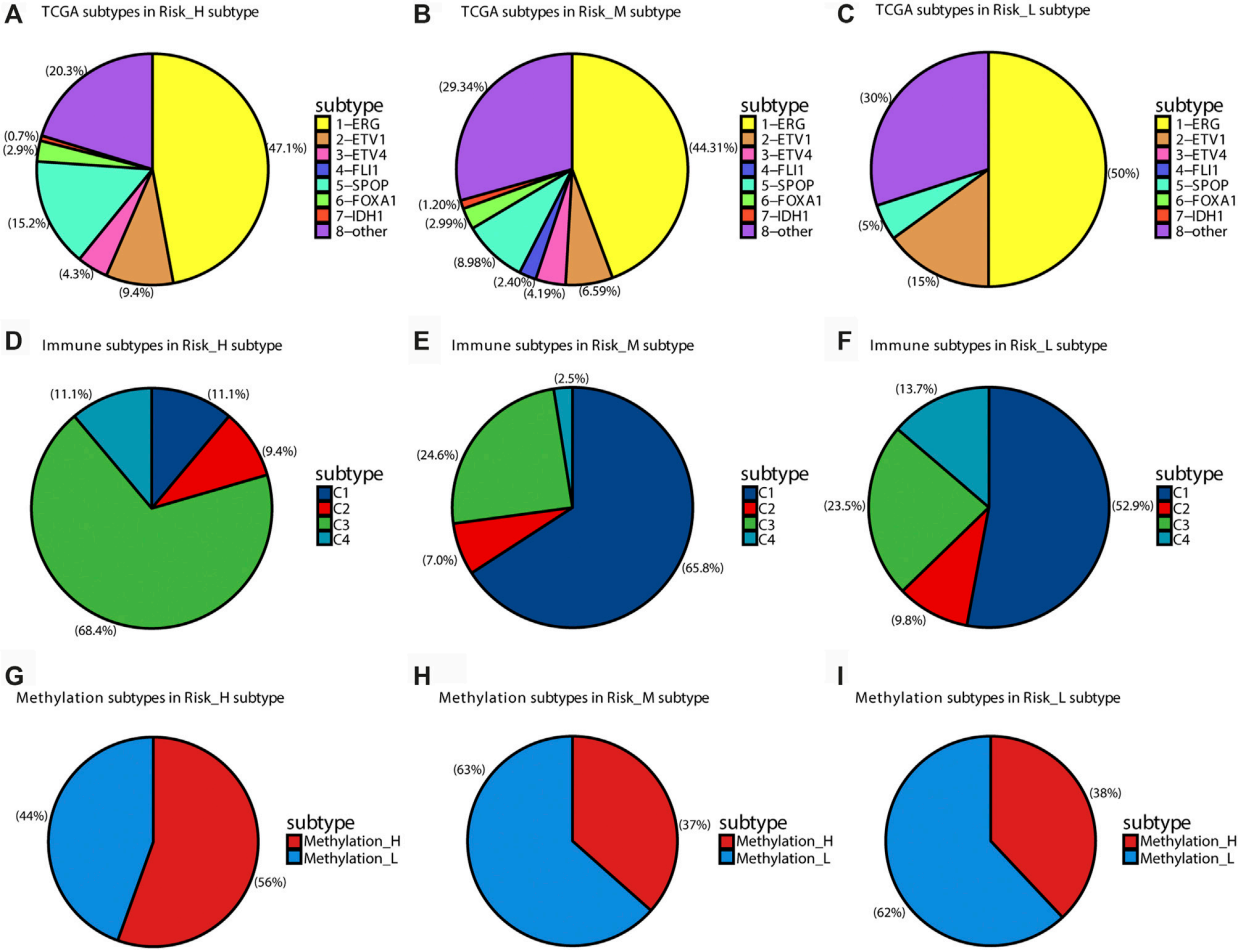
The distribution of other TCGA subtypes in subtypes of this research. **(A)** The distribution of TCGA subtypes in Risk_H subtype. **(B)** The distribution of TCGA subtypes in Risk_M subtype. **(C)** The distribution of TCGA subtypes in Risk_L subtype. **(D)** The distribution of immune subtypes in Risk_H subtype. **(E)** The distribution of immune subtypes in Risk_M subtype. **(F)** The distribution of immune subtypes in Risk_L subtype. **(G)** The distribution of DNA methylation subtypes in Risk_H subtype. **(H)** The distribution of DNA methylation subtypes in Risk_M subtype. **(I)** The distribution of DNA methylation subtypes in Risk_L subtype.

Seven subtypes were previously defined based on ETS fusions or mutations in *SPOP*, *FOXA1*, and *IDH1* (Abeshouse et al., 2015). We found that *SPOP mutations* were overrepresented in the Risk_H subtype (chi-square test *p* < 0.05, Figures 3A–C). Furthermore, we found that the Risk_L subtype had lower heterogeneity than other subtypes, including only four subtypes (Figure 3C). Furthermore, based on previously established immune-based subtypes, we found that the Risk_H subtype was mainly composed of the C1 subtype (Zhang et al., 2020), which is associated with a poor prognosis; however, Risk_M and Risk_L subtypes were mainly composed of the C3 subtype, associated with a relatively favorable prognosis (Figures 3D–F). In addition, based on subtypes differing in methylation patterns (Zhang et al., 2021), we found that the Risk_H subtype was mainly composed of the Methylation_H subtype, associated with a poor prognosis; however, Risk_M and Risk_L subtypes were mainly composed of the Methylation_L subtype, associated with a better prognosis (Figures 3G–I). Taken together, *SPOP* mutations, which are associated with a poor prognosis in PCa, were positively correlated with the Risk_H subtype; the *SPOP* mutation frequency in our subtypes decreased in the following order: Risk_H > Risk_M > Risk_L. Our previous studies all support the poor prognostic characteristics of the Risk_H subtype.

### Risk_H Subtype Shows Greater Tumor Purity and Less Immune Cell Infiltration

**FIGURE 4:**
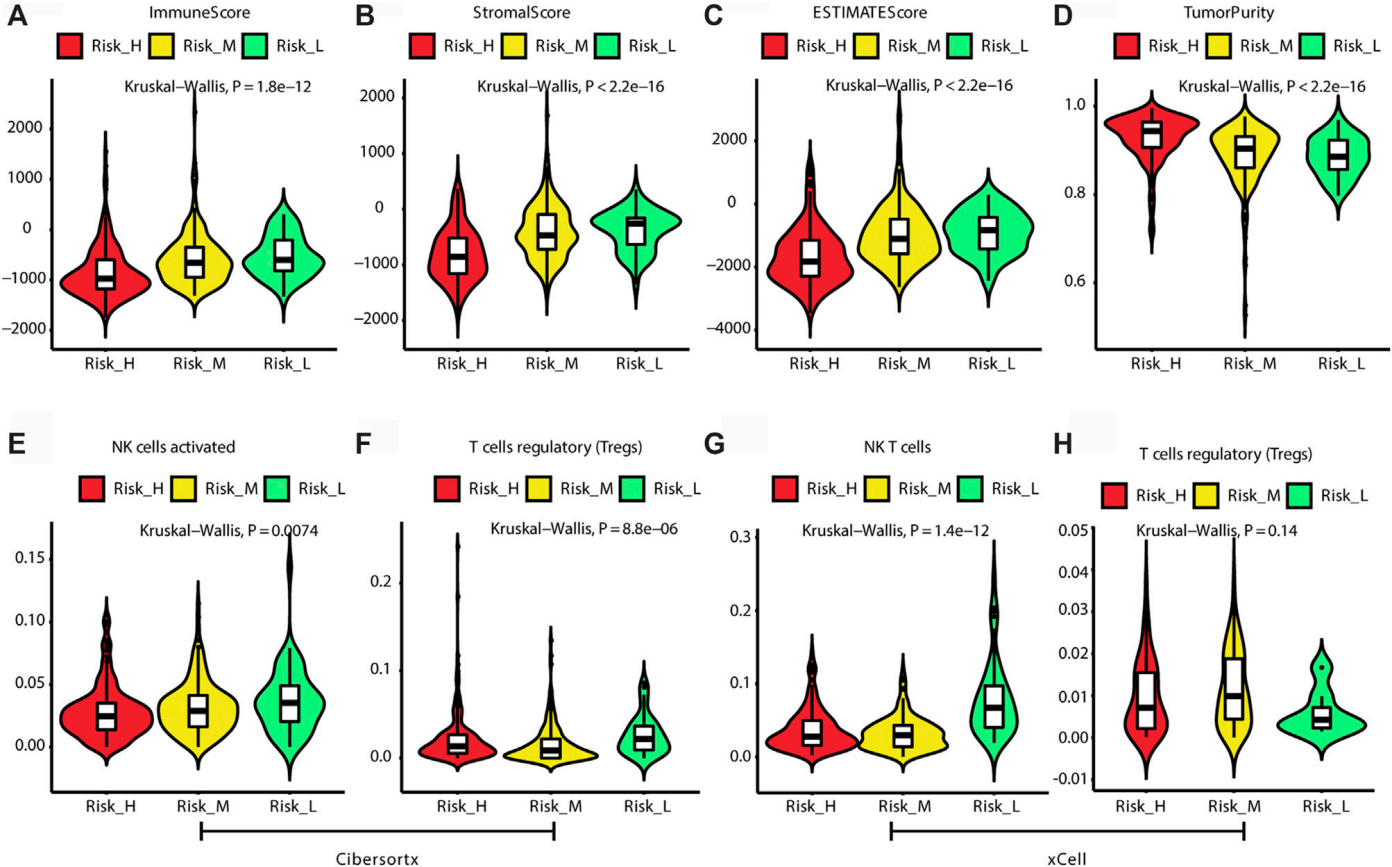
The difference of tumor microenvironment among three subtypes. **(A)** The violin plot shows immune score is the lowest in Risk_H subtype. **(B)** The violin plot shows stromal score is the lowest in Risk_H subtype. **(C)** The violin plot shows ESTIMATE score is the lowest in Risk_H subtype. **(D)** The violin plot shows tumor purity is the highest in Risk_H subtype. **(E)** The violin plot shows content of NK cells activated is the lowest in Risk_H subtype. **(F)** The violin plot shows content of Treg cells is the lowest in Risk_H subtype. **(G)** The violin plot shows content of NK T cells is the lowest in Risk_H subtype. **(H)** The violin plot shows content of Treg cells is not significantly different among subtypes. (*p* < 0.05 is defined as statistically significant).

According to the ESTIMATE algorithm, the Risk_H subtype had lower immune, stromal, and ESTIMATE scores (Figures 4A,B) and a higher tumor purity (Figure 4D). Based on the CIBERSORTx algorithm, which predicts the immune cell composition in the tumor microenvironment based on gene expression data for 22 kinds of immune cells, we found that activated NK cells and regulatory T cells (Tregs) were significantly less frequent in the Risk_H type than in the other types (Kruskal–Wallis test *p* < 0.05, Figures 4E,F). According to the xCell algorithm, we found that NK T cells were also significantly less frequent in the Risk_H type than in the other types (Kruskal–Wallis test *p* < 0.05, Figure 4G). However, we found that the frequency of Tregs did not differ significantly among subtypes (Kruskal–Wallis test *p* > 0.05, Figure 4H). Collectively, these findings indicated that the Risk_H subtype had greater tumor purity and a smaller immune cell component. With respect to immune cell infiltration, activated NK cells and Tregs were reduced in the Risk_H type.

### Mutational Landscape Across the Newly Established Risk Subtypes

**FIGURE 5:**
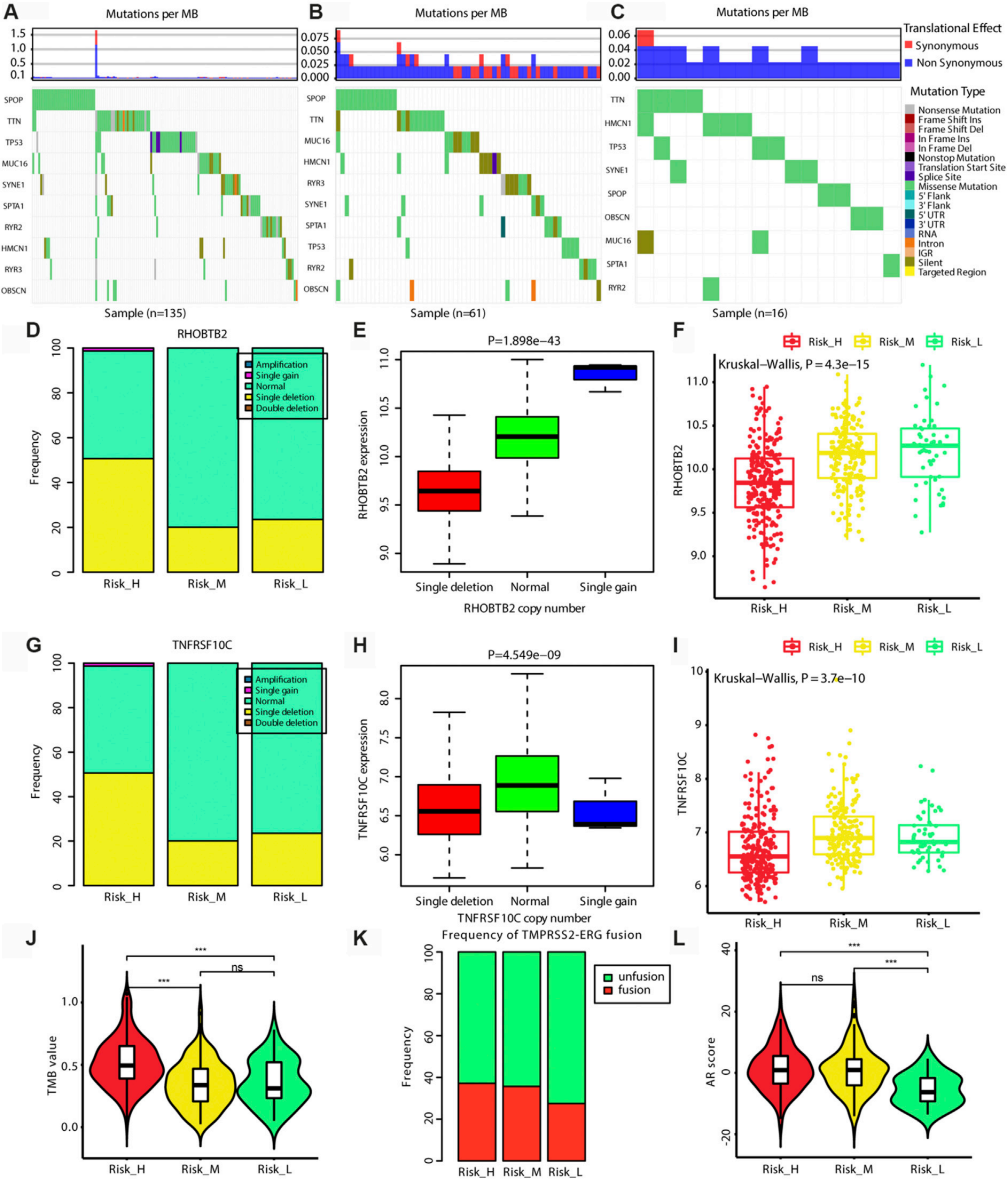
The genomic differences among three subtypes. **(A)** The map of waterfall for the Risk_H subtype. **(B)** The map of waterfall for the Risk_M subtype. **(C)** The map of waterfall for the Risk_L subtype. **(D)** The frequency of CNV in *RHOBTB2* in Risk_H subtype is significantly higher than that in other subtype. **(E)** The expression level of *RHOBTB2* is significantly correlated with its CNV, and the expression level of *RHOBTB2* is decreased with single deletion. **(F)** The expression level of *RHOBTB2* is the lowest in Risk_H subtype. **(G)** The frequency of CNV in *TNFRSF10C* in Risk_H subtype is significantly higher than that in other subtype. **(H)** The expression level of *TNFRSF10C* is significantly correlated with its CNV, and the expression level of *TNFRSF10C* is decreased with single deletion. **(I)** The expression level of *TNFRSF10C* is the lowest in Risk_H subtype. **(J)** The violin plot shows TMB values of patients are the highest in Risk_H subtype. Wilcoxon *p* values were calculated. **(K)** The bar graph shows TMPRSS2−ERG fusion status among three subtypes. **(L)** The violin plot shows AR scores of patients are the higher in Risk_H and Risk_M subtypes. Wilcoxon *p* values were calculated. (PCa, prostate cancer; CNV, copy number variation; AR, androgen receptor. And *p* < 0.05 is defined as statistically significant).

As shown in Figures 5A–C, we found that the frequencies of SNVs in *SPOP* were higher in Risk_H and Risk_M than in Risk_L (chi-squared test *p* < 0.05). More single-copy deletion events were observed in the Risk_H subtype for *RHOBTB2* and *TNFRSF10C* (chi-squared test *p* < 0.05). As shown in Figures 5E,H, the single deletions of *RHOBTB2* and *TNFRSF10C* were associated with lower expression levels (Kruskal–Wallis test *p* < 0.05). Consistent with these findings, lower expression levels of *RHOBTB2* and *TNFRSF10C* were detected in the Risk_H subtype (Kruskal–Wallis test *p* < 0.001, Figures 5F,I). Furthermore, as shown in Figure 5J, TMB values for patients were highest in the Risk_H subtype. Although the frequency of the TMPRSS2-ERG fusion did not differ significantly among subtypes, it was highest in the Risk_H subtype (Figure 5K). Additionally, AR scores for patients were higher in the Risk_H and Risk_M subtypes than in the Risk-L subtype (Figure 5L, Wilcoxon *p* < 0.001). These data suggest that SNVs in *SPOP* and CNVs in *RHOBTB2* and *TNFRSF10C* are more common in the Risk_H subtype than in other subtypes. Low expression levels of *RHOBTB2* and *TNFRSF10C* in Risk_H could be associated with single deletion CNV events.

### Identification of a Single Gene Interaction Network by WGCNA Associated With the Risk_H Subtype

**FIGURE 6:**
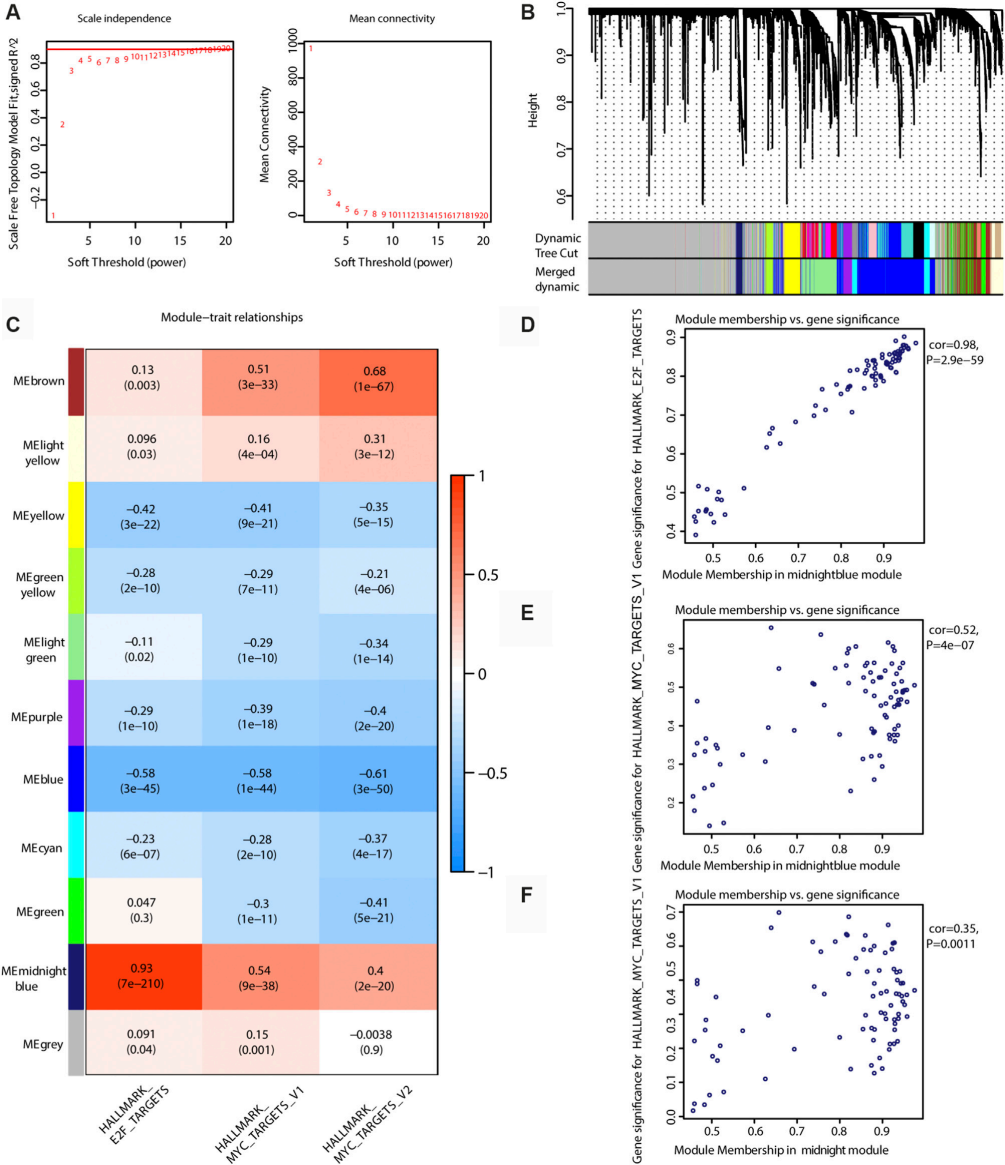
WGCNA to find the genes for the development of the model. **(A)** The relationship of soft threshold and TOM-based dissimilarity **(left)**. The relationship of soft threshold and mean connectivity **(right)**. **(B)** After the dynamic of cut and merged, a total of 11 gene modules were finally generated. **(C)** Heat map for the correlation of gene modules and phenotypes. **(D)** The scatter plot shows the gene significance for E2F ssGSEA score is correlated with module membership in midnightblue module (Pearson rho = 0.98, *p* < 0.01). **(E)** The scatter plot shows the gene significance for MYC_V2 ssGSEA score is correlated with module membership in midnightblue module (Pearson rho = 0.35, *p* < 0.01). (WGCNA, weighted correlation network analysis; TOM, topological overlap matrix. And *p* < 0.05 is defined as statistically significant).

As shown in Figure 6A, the soft threshold value was set to 8. Eleven gene interaction networks were finally defined (Figure 6B). The midnight blue network shown in Figure 6C was mostly correlated with ssGSEA scores for E2F and MYC, indicating that this gene network best represents the Risk_H subtype. As shown in Figures 6D–F, values for gene significance and module membership were significantly associated (E2F: rho = 0.98, *p* < 0.001; MYC_V1: rho = 0.52, *p* < 0.001; MYC_V2: rho = 0.35, *p* = 0.0011). Taken together, we identified a single group of genes that effectively reflects the Risk_H subtype.

### Construction of a Prognostic Model Consisting of 12 Genes

**FIGURE 7:**
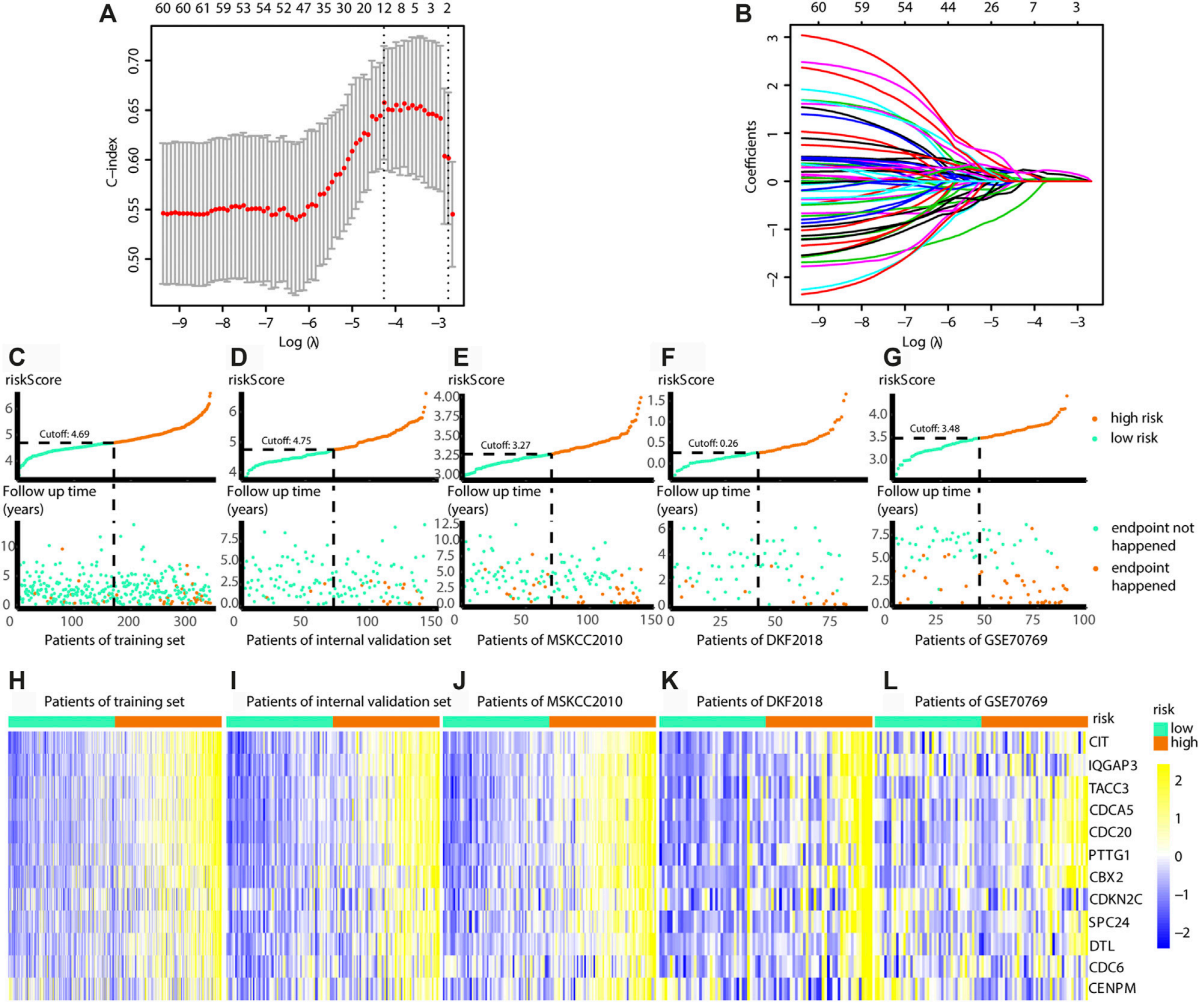
Build the risk model by LASSO. **(A)** Cross validation based on C-index to determine the best choice of genes in the model. **(B)** Genes in the different combinations and their corresponding coefficients. **(C)** Patients of training set are arranged in the same ascending order of the risk score. **(D)** Patients of internal validation set are arranged in the same ascending order of the risk score. **(E)** Patients of MSKCC2010 data set are arranged in the same ascending order of the risk score. **(F)** Patients of DKF2018 data set are arranged in the same ascending order of the risk score. **(G)** Patients of GSE70769 data set are arranged in the same ascending order of the risk score. **(C**–**G)** Patients are divided into different risk levels according to the median of the risk scores in their respective data sets **(upward)**. The relationship between the survival outcome and risk levels of patients. Low-risk patients are shown on the left side of the dotted line and high-risk patients are shown on the right side **(downward)**. **(H**–**L)** Heatmaps show the expression levels of genes in the model, and indicate the model is robust in these data sets. (LASSO, least absolute shrinkage and selection operator. And *p* < 0.05 is defined as statistically significant).

Since the Risk_H subtype was associated with a poor prognosis, the midnight gene network was chosen to train a prognostic model *via* LASSO. In the training group, one 12-gene combination had the highest cross-validated C-index (Figure 7A). Changes in gene coefficients during the selection procedure are shown in Figure 7B. Risk scores were obtained for patients as follows: $Risk\;score=\sum_{n=1}^{12}\left(coefficient_{n}\times {\text{expression of gene}}_{\text{n}}\right)$. The coefficients for each gene are given in Table 3. Subsequently, patients in the training group, internal validation group, and three external validation groups (GSE70769, DKF 2018, and MSKCC 2010) were ranked in ascending order based on risk scores. Due to the batch effect across platforms, the median risk score in each group was selected as the cut-off value to divide patients into high-risk and low-risk groups (Figures 7C–G). We found that patients identified as high risk had a poorer prognosis than patients identified as low risk. The global expression levels of the 12 genes are shown in Figures 7H–L. Collectively, we developed a 12-gene prognostic model with robust global expression levels across all data sets.

**TABLE 3:** LASSO coefficients of genes in model.

Gene name	Model coefficient
CIT	0.172
IQGAP3	0.045
TACC3	0.185
CDCA5	0.007
CDC20	0.24
PTTG1	0.193
CBX2	0.177
CDKN2C	0.017
SPC24	0.009
DTL	−0.026
CDC6	−0.022
CENPM	−0.451

### Validation of the Model Accuracy

**FIGURE 8:**
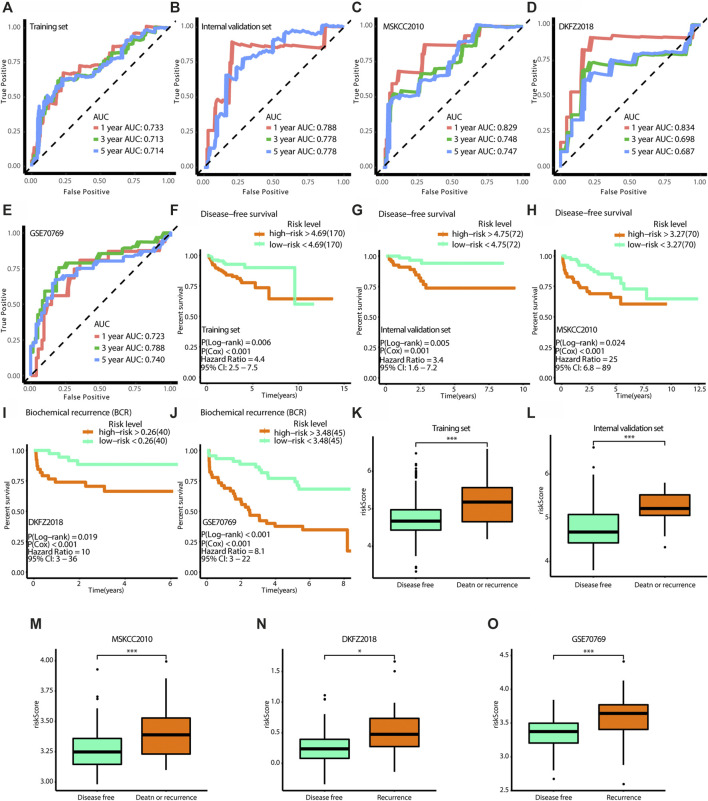
Verification of the effectiveness of the model. **(A**–**E)** The ROC curves of 1-year, 3-year, and 5-year follow-up time. **(F**–**J)** Kaplan-Meier curves for survival analysis. **(K**–**O)** The boxplots show the difference of risk score between survival outcomes. **(A**,**F**,**K)** The results in the training set. **(B**,**G**,**L)** The results in the internal validation set. **(C**,**H**,**M)** The results in MSKCC 2010. **(D**,**I**,**N)** The results in DKFZ 2018. **(E**,**J**,**O)** The results in GSE70769. (AUC, area under curve. * means *p* < 0.05, ** means *p* < 0.01, *** means *p* < 0.001, ns means *p* > 0.05, and *p* < 0.05 is defined as statistically significant. And *p* < 0.05 is defined as statistically significant).

To validate the accuracy of the model, a tdROC analysis was performed. In the training group, 1°year AUC = 0.733, 3°year AUC = 0.713, and 5°year AUC = 0.714 (Figure 8A). In the internal validation group, 1°year AUC = 0.788, 3°year AUC = 0.778, 5°year AUC = 0.778 (Figure 8B); in the MSKCC2010 data set, 1°year AUC = 0.829, 3°year AUC = 0.748, 5°year AUC = 0.747 (Figure 8C); in the DKF2018 data set, 1°year AUC = 0.834, 3°year AUC = 0.698, 5°year AUC = 0.687 (Figure 8D); in the GSE70769 data set, 1°year AUC = 0.723, 3°year AUC = 0.788, 5°year AUC = 0.740 (Figure 8E). Subsequently, we found that the high-risk patients identified by this model had worse survival in the training group (log-rank test *p* = 0.006, Cox test *p* < 0.001), internal validation group (log-rank test *p* = 0.005, Cox test *p* = 0.001), MSKCC2010 data set (log-rank test *p* = 0.024, Cox test *p* < 0.001), DKF2018 data set (log-rank test *p* = 0.019, Cox test *p* < 0.001), and GSE70769 data set (log-rank test *p* < 0.001, Cox test *p* < 0.001), as shown in Figures 8F–J. Furthermore, we found that patients who died or experienced recurrence had higher risk scores in the training group (Wilcoxon test *p* < 0.001), internal validation group (Wilcoxon test *p* < 0.001), MSKCC2010 data set (Wilcoxon test *p* < 0.001), DKF2018 data set (Wilcoxon test *p* < 0.05), and GSE70769 data set (Wilcoxon test *p* < 0.001). According to univariate and multivariate Cox regression analyses (Table 4), this model and the Gleason grade were independent predictors of prognosis. Taken together, the prognostic model had high accuracy.

**TABLE 4:** Results of univariate and multivariate Cox regression.

	Univariate analysis		Multivariate analysis	
	HR (95% CI)	*p* value	HR (95% CI)	*p* value
Race	0.787 (0.305–2.03)	0.62		
Age	0.998 (0.945–1.053)	0.931		
Pathological N	1.366 (0.643–2.903)	0.417		
Pathological T	4.279 (1.364–13.426)	0.013	1.113 (0.308–4.018)	0.87
Gleason grade	2.143 (1.42–3.235)	<0.001	1.792 (1.129–2.844)	0.013
Prior malignancy	0.767 (0.104–5.638)	0.794		
Diagnostic CT or MRI	1.38 (0.954–1.996)	0.087		
Residual tumor	1.272 (0.897–1.806)	0.177		
PSA	1.012 (0.991–1.034)	0.255		
Risk score	3.423 (1.862–6.293)	<0.001	2.099 (1.043–4.224)	0.038

PSA, prostate-specific antigen; and *p* < 0.05 is defined as statistically significant.

### Target Drug Prediction for High-Risk Patients

**FIGURE 9:**
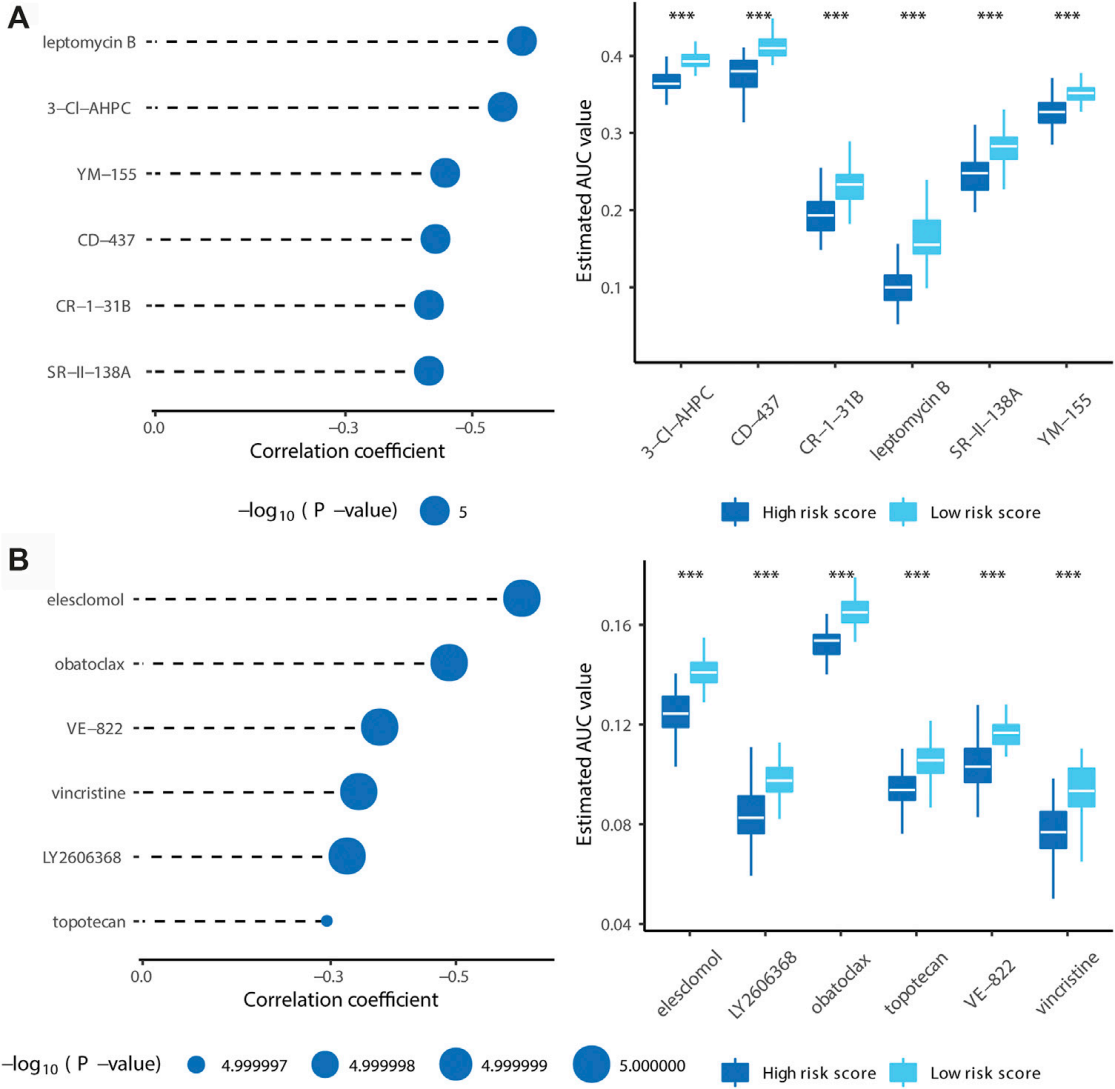
Identification of candidate agents with higher drug sensitivity in patients with high risk score. **(A)** The results of Spearman’s correlation analysis and differential drug response analysis of six CTRP-derived compounds. **(B)** The results of Spearman’s correlation analysis and differential drug response analysis of six PRISM-derived compounds. Note that lower values on the y-axis of boxplots imply greater drug sensitivity. (* means *p* < 0.05, ** means *p* < 0.01, *** means *p* < 0.001, ns means *p* > 0.05, and *p* < 0.05 is defined as statistically significant. And *p* < 0.05 is defined as statistically significant).

Using compounds from the CTRP and PRISM databases, we predicted drug sensitivity for patients with high risk scores. As shown in Figure 9A, 3-CI-AHPC, CD-437, CR-1-31B, leptomycin B, SR-II-138A, and YM-155 sensitivities were high for patients with high risk scores (Spearman correlation rho < −0.3, Spearman correlation test *p* < 0.001, and Wilcoxon test *p* < 0.001). As shown in Figure 9B, elesclomol, LY2606368, obatoclax, topotecan, VE-822, and vincristine sensitivities were high for patients with high risk scores (Spearman correlation test rho < −0.3, Spearman correlation test *p* < 0.001, and Wilcoxon test *p* < 0.001). Collectively, we identified ten target drugs predicted to be effective for high-risk patients, and leptomycin B, LY2606368, and vincristine showed particularly high effectiveness.

## Discussion

The mechanism underlying PCa progression is complex and cannot be explained by a single pathway. Accordingly, in this study, we used gene expression information for eight PCa-related pathways (i.e., hypoxia, androgen response, PI3K-AKT-mTOR signaling, E2F targets, MYC targets V1, MYC targets V2, glycolysis, fatty acid metabolism, and oxidative phosphorylation pathways) extracted from Molecular Signatures Database v7.2 and data for PCa cohorts from multiple platforms (TCGA, GSE70769, DKF 2018, and MSKCC 2010) to identify three PCa subtypes (Risk_H, Risk_M and Risk_L). These subtypes were then used to construct a risk-predicting model and drug sensitivity prediction was performed for the high-risk group.

For patients with PCa, an elevated hypoxic status is related to a more aggressive and advanced disease; hypoxia reduction could increase immunity and the response to specific immunotherapies (Jayaprakash et al., 2018). Additionally, prostate is an androgen-dependent organ, and androgen interactions with androgen receptors play a key role in the progression of PCa. Endocrine therapy in PCa is aimed at lowering serum androgen levels and inhibiting androgen receptor; when this approach fails, PCa advances to a hormone-resistant state (Heinlein and Chang, 2004; Shafi et al., 2013). The PI3K-AKT-mTOR pathway interacts with multiple cellular cascades, further promoting PCa progression and aggression, and drugs targeting this pathway are employed in clinical settings (Shorning et al., 2020). E2F and MYC synergistically contribute to cell cycle regulation and are involved in tumorigenesis (Liu et al., 2015). Metabolic adaptation is pivotal for malignancy given the high energy demand, and glycolytic, fatty acid biosynthesis, and oxidative phosphorylation contribute to proliferation and worse outcomes in PCa (Schöpf et al., 2016; Xiao et al., 2018; Balusamy et al., 2020). We classified samples into three subtypes with different patterns of pathway enrichment.

Among the three subtypes, the cluster with enrichment for the E2F and MYC pathways was identified as high-risk group (Risk_H), which was associated with the worst clinical outcomes. Further analyses of the proliferation scores and *MKI67* gene expression level support the highly proliferative feature of the Risk_H cohort. Additionally, the proportions of immune and stromal cells were highest in the Risk_L cohort. NK cells, which possesses important anti-cancer functions (Abel et al., 2018), were most abundant in the Risk_L group. Thus, the poor prognosis in the Risk_H group can be explained from the perspective of immune activity. Additionally, *RhoBTB2*, a candidate tumor suppressor, has been implicated in various cancers, such as breast cancer and lung cancer (McKinnon et al., 2008). However, little is known about its role in PCa. We found that the single copy deletion of *RhoBTB2* was most frequent in the Risk_H group, while its overall expression was highest in the Risk_L group. This finding may provide an entry point for future PCa research. The *TNFRSF10C* gene, also known as *TRAIL-R3*, is a decoy receptor for tumor necrosis factor-related apoptosis-inducing ligand, inducing tumor apoptosis in multiple malignancies (Almodóvar et al., 2004). We detected copy number variation distinguishing the Risk_H and Risk_L subtypes, and this may further explain the poor prognosis in the Risk_H group.

After establishing the prognostic value and properties of the subtypes, we constructed a twelve gene-based risk-prediction model. This model could supplement current strategies for clinical decision-making and prognostic predictions. Finally, we filtered twelve drugs expected to show high sensitivity in high-risk patients with PCa, 3-CI-HPC, CD-437, CR-1-31B, leptomycin B, SR-II-138A, YM-155, elesclomol, LY2606368, obatoclax, topotecan, VE-822, and vincristine.

As a complex and heterogeneous disease, PCa is difficult to manage by a universal treatment approach. In this study, we divided PCa into three clusters based on eight pivotal pathways, allowing for more innovative and objective results than those obtained by analyses of single pathways. Moreover, we translated the results into a clinically useful tool and identified potentially effective drugs for high-risk patients, providing direct guidance for clinical strategies aimed at precision medicine. However, our study had limitations. First, the results are based on retrospective investigations of cohorts from multiple platforms; prospective explorations are needed to validate our results. Further clinical studies of the drug candidates are needed. Despite these drawbacks, our results provide novel ideas for PCa management.

## Data Availability

The original contributions presented in the study are included in the article/Supplementary Material, further inquiries can be directed to the corresponding author.
