# Supplementary material for: Establishment of Novel Prostate Cancer Risk Subtypes and A Twelve-Gene Prognostic Model
Source: Front Mol Biosci. 2021 May 28;8:676138. doi: 10.3389/fmolb.2021.676138 (PMC8193735; doi:10.3389/fmolb.2021.676138)
Supplement: Supplementary file 1 [file DataSheet1.PDF]

# *Supplementary Material*

## 1 Supplementary Figure 1.

**(A-C)** For different k values, the figure reflectes the item-consensus of each patient in different clusters. The item-consensus reflects the degree of representation of an individual to different clusters. The greater the value, the more representative the individual is of the characteristics of the corresponding cluster. **(D)** The x-coordinate of the graph is items, and the y-coordinate is the value of k. Each color corresponds to different cluster classification colors in the consensus cluster. If items always change the type of cluster (i.e. change the color in a column) it indicates an unstable classification relationship. If a cluster has a large number of samples with unstable classification, it indicates that the cluster is not a stable classification and cannot become a subtyping. **(E)** The diagram shows the cluster-consensus for each cluster with different k values. The cluster-consensus refers to the average value of the consensus matrix of each cluster, and represents the degree of consensus of this cluster. The higher the cluster-consensus of the cluster, the higher the stability of this cluster.

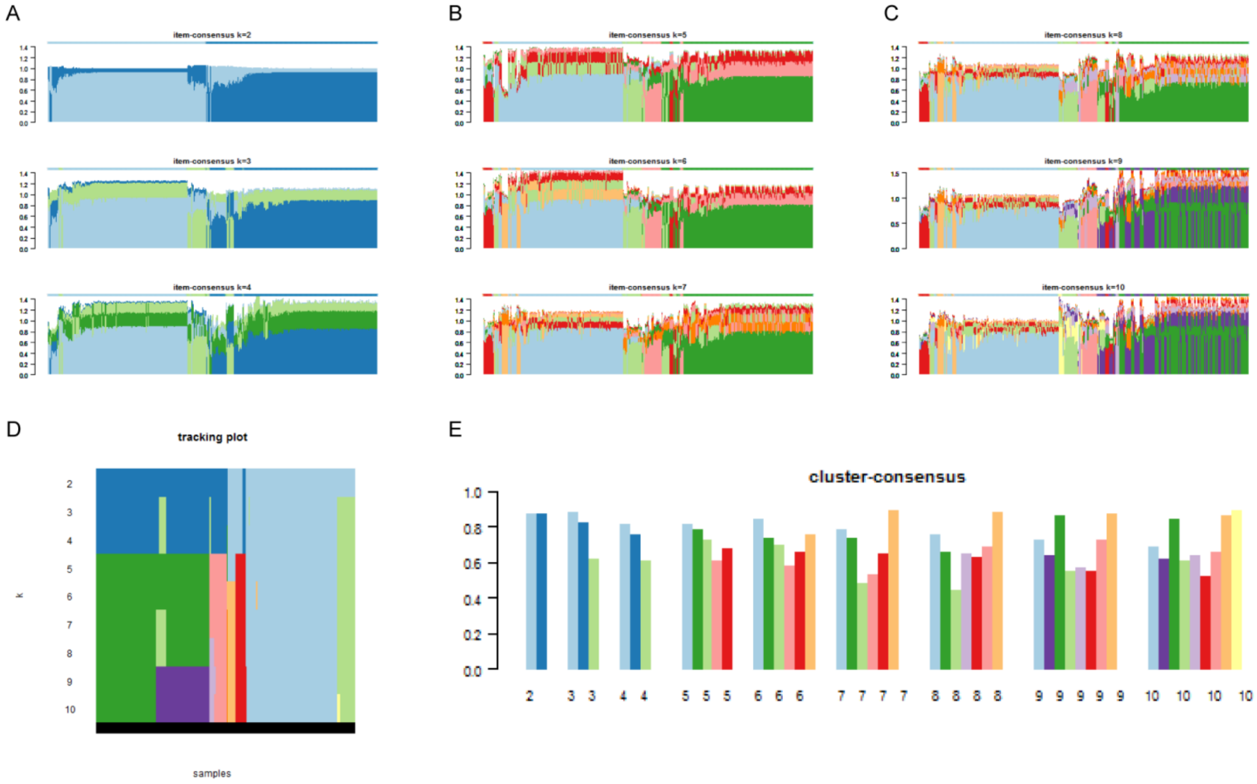


## 2 Supplementary Table 1.

Supplementary Table 1: Clinical outcomes for 484 PCa patients in TCGA.

| patient_id | DFS | DFS.time | PFI | PFI.time |
|---|---|---|---|---|

| | | | | |
|---|---|---|---|---|
| TCGA-2A-A8VL | 0 | 1.701369863 | 0 | 1.701369863 |
| TCGA-2A-A8VO | 0 | 4.660273973 | 0 | 4.660273973 |
| TCGA-2A-A8VT | 0 | 3.761643836 | 0 | 3.761643836 |
| TCGA-2A-A8VV | 0 | 1.838356164 | 0 | 1.838356164 |
| TCGA-2A-A8VX | 0 | 3.775342466 | 0 | 3.775342466 |
| TCGA-2A-A8W1 | 0 | 0.306849315 | 0 | 0.306849315 |
| TCGA-2A-A8W3 | 1 | 0.542465753 | 1 | 0.542465753 |
| TCGA-2A-AAYF | 0 | 3.736986301 | 0 | 3.736986301 |
| TCGA-2A-AAYO | 0 | 3.484931507 | 0 | 3.484931507 |
| TCGA-2A-AAYU | 0 | 1.684931507 | 0 | 1.684931507 |
| TCGA-4L-AA1F | 0 | 0.95890411 | 0 | 0.95890411 |
| TCGA-CH-5737 | 0 | 0.249315068 | 0 | 0.249315068 |
| TCGA-CH-5738 | 0 | 0.580821918 | 0 | 0.580821918 |
| TCGA-CH-5739 | 0 | 1.838356164 | 0 | 1.838356164 |
| TCGA-CH-5740 | 0 | 0.084931507 | 0 | 0.084931507 |
| TCGA-CH-5741 | 0 | 1.082191781 | 0 | 1.082191781 |
| TCGA-CH-5743 | 0 | 1.164383562 | 1 | 1.164383562 |
| TCGA-CH-5744 | 0 | 0.164383562 | 0 | 0.164383562 |
| TCGA-CH-5745 | 0 | 0.249315068 | 0 | 0.249315068 |
| TCGA-CH-5746 | 0 | 2.002739726 | 0 | 2.002739726 |
| TCGA-CH-5748 | 0 | 0.084931507 | 0 | 0.084931507 |
| TCGA-CH-5750 | 0 | 1.084931507 | 0 | 1.084931507 |
| TCGA-CH-5751 | 0 | 2.917808219 | 1 | 1 |
| TCGA-CH-5752 | 0 | 2.583561644 | 0 | 2.583561644 |
| TCGA-CH-5753 | 0 | 0.084931507 | 0 | 0.084931507 |
| TCGA-CH-5754 | 0 | 0.169863014 | 0 | 0.169863014 |
| TCGA-CH-5762 | 0 | 3.668493151 | 0 | 3.668493151 |
| TCGA-CH-5763 | 0 | 1 | 0 | 1 |
| TCGA-CH-5764 | 0 | 0.084931507 | 0 | 0.084931507 |
| TCGA-CH-5765 | 0 | 1.917808219 | 0 | 1.917808219 |
| TCGA-CH-5766 | 0 | 0.084931507 | 0 | 0.084931507 |
| TCGA-CH-5767 | 0 | 1.254794521 | 0 | 1.254794521 |
| TCGA-CH-5768 | 0 | 2.002739726 | 0 | 2.002739726 |
| TCGA-CH-5769 | 0 | 0.169863014 | 0 | 0.169863014 |
| TCGA-CH-5771 | 0 | 1.084931507 | 0 | 1.084931507 |
| TCGA-CH-5772 | 0 | 1.331506849 | 0 | 1.331506849 |
| TCGA-CH-5788 | 1 | 2.153424658 | 0 | 2.249315068 |
| TCGA-CH-5789 | 0 | 0.832876712 | 0 | 0.832876712 |
| TCGA-CH-5790 | 0 | 2.668493151 | 0 | 2.668493151 |
| TCGA-CH-5791 | 0 | 2.750684932 | 1 | 1.084931507 |
| TCGA-CH-5792 | 0 | 0.249315068 | 0 | 0.249315068 |
| TCGA-CH-5794 | 0 | 2.416438356 | 0 | 2.416438356 |
| TCGA-EJ-5494 | 0 | 4.046575342 | 0 | 4.046575342 |
| TCGA-EJ-5495 | 0 | 4.821917808 | 0 | 4.821917808 |
| TCGA-EJ-5498 | 0 | 4.712328767 | 0 | 4.712328767 |

| | | | | |
|---|---|---|---|---|
| TCGA-EJ-5499 | 0 | 3.876712329 | 0 | 3.876712329 |
| TCGA-EJ-5501 | 0 | 3.739726027 | 0 | 3.739726027 |
| TCGA-EJ-5502 | 0 | 3.208219178 | 0 | 3.208219178 |
| TCGA-EJ-5503 | 0 | 3.810958904 | 0 | 3.810958904 |
| TCGA-EJ-5504 | 1 | 0.210958904 | 0 | 3.909589041 |
| TCGA-EJ-5505 | 0 | 2.526027397 | 0 | 2.526027397 |
| TCGA-EJ-5506 | 0 | 3.783561644 | 0 | 3.783561644 |
| TCGA-EJ-5507 | 0 | 3.991780822 | 0 | 3.991780822 |
| TCGA-EJ-5508 | 0 | 5.383561644 | 0 | 5.383561644 |
| TCGA-EJ-5509 | 0 | 4.010958904 | 0 | 4.010958904 |
| TCGA-EJ-5510 | 0 | 5.169863014 | 0 | 5.169863014 |
| TCGA-EJ-5511 | 0 | 4.043835616 | 0 | 4.043835616 |
| TCGA-EJ-5512 | 0 | 4.747945205 | 0 | 4.747945205 |
| TCGA-EJ-5514 | 0 | 5.010958904 | 0 | 5.010958904 |
| TCGA-EJ-5515 | 0 | 5.019178082 | 0 | 5.019178082 |
| TCGA-EJ-5516 | 0 | 5.175342466 | 0 | 5.175342466 |
| TCGA-EJ-5517 | 0 | 5.175342466 | 0 | 5.175342466 |
| TCGA-EJ-5518 | 0 | 5.802739726 | 1 | 5.764383562 |
| TCGA-EJ-5519 | 1 | 0.230136986 | 0 | 5.375342466 |
| TCGA-EJ-5521 | 0 | 6.243835616 | 0 | 6.243835616 |
| TCGA-EJ-5522 | 0 | 5.695890411 | 0 | 5.695890411 |
| TCGA-EJ-5524 | 0 | 5.156164384 | 1 | 0.726027397 |
| TCGA-EJ-5525 | 1 | 0.926027397 | 1 | 1.495890411 |
| TCGA-EJ-5526 | 0 | 5.230136986 | 1 | 1.471232877 |
| TCGA-EJ-5527 | 0 | 4.871232877 | 0 | 4.871232877 |
| TCGA-EJ-5530 | 0 | 5.019178082 | 0 | 5.019178082 |
| TCGA-EJ-5531 | 0 | 3.487671233 | 0 | 3.487671233 |
| TCGA-EJ-5532 | 0 | 5.008219178 | 0 | 5.008219178 |
| TCGA-EJ-5542 | 0 | 4.145205479 | 0 | 4.145205479 |
| TCGA-EJ-7115 | 0 | 7.361643836 | 0 | 7.361643836 |
| TCGA-EJ-7123 | 0 | 7.046575342 | 0 | 7.046575342 |
| TCGA-EJ-7125 | 0 | 7.808219178 | 0 | 7.808219178 |
| TCGA-EJ-7218 | 0 | 6.964383562 | 0 | 6.964383562 |
| TCGA-EJ-7314 | 0 | 3.150684932 | 0 | 3.150684932 |
| TCGA-EJ-7315 | 0 | 2.761643836 | 0 | 2.761643836 |
| TCGA-EJ-7317 | 0 | 2.112328767 | 0 | 2.112328767 |
| TCGA-EJ-7318 | 1 | 0.517808219 | 1 | 1.04109589 |
| TCGA-EJ-7321 | 0 | 2.257534247 | 0 | 2.257534247 |
| TCGA-EJ-7325 | 0 | 2.931506849 | 0 | 2.931506849 |
| TCGA-EJ-7327 | 0 | 2.528767123 | 0 | 2.528767123 |
| TCGA-EJ-7328 | 0 | 2.306849315 | 0 | 2.306849315 |
| TCGA-EJ-7330 | 0 | 0.523287671 | 0 | 0.523287671 |
| TCGA-EJ-7331 | 1 | 0.517808219 | 0 | 2.032876712 |
| TCGA-EJ-7781 | 0 | 2.939726027 | 0 | 2.939726027 |
| TCGA-EJ-7782 | 0 | 3.197260274 | 0 | 3.197260274 |
| TCGA-EJ-7783 | 1 | 0.221917808 | 0 | 2.156164384 |

| | | | | |
|---|---|---|---|---|
| TCGA-EJ-7784 | 0 | 2.139726027 | 0 | 2.139726027 |
| TCGA-EJ-7785 | 0 | 3.224657534 | 0 | 3.224657534 |
| TCGA-EJ-7786 | 0 | 3.002739726 | 0 | 3.002739726 |
| TCGA-EJ-7788 | 0 | 2.353424658 | 0 | 2.353424658 |
| TCGA-EJ-7789 | 0 | 3.02739726 | 0 | 3.02739726 |
| TCGA-EJ-7791 | 0 | 3.063013699 | 0 | 3.063013699 |
| TCGA-EJ-7792 | 0 | 3.824657534 | 0 | 3.824657534 |
| TCGA-EJ-7793 | 0 | 0.312328767 | 0 | 0.312328767 |
| TCGA-EJ-7794 | 0 | 3.578082192 | 0 | 3.578082192 |
| TCGA-EJ-7797 | 0 | 2.693150685 | 0 | 2.693150685 |
| TCGA-EJ-8468 | 0 | 6.430136986 | 0 | 6.430136986 |
| TCGA-EJ-8469 | 1 | 5.273972603 | 1 | 5.273972603 |
| TCGA-EJ-8470 | 0 | 3.175342466 | 0 | 3.175342466 |
| TCGA-EJ-8472 | 1 | 0.536986301 | 1 | 0.536986301 |
| TCGA-EJ-8474 | 0 | 2.098630137 | 0 | 2.098630137 |
| TCGA-EJ-A46B | 0 | 1.8 | 0 | 1.8 |
| TCGA-EJ-A46D | 0 | 1.715068493 | 0 | 1.715068493 |
| TCGA-EJ-A46E | 0 | 1.435616438 | 0 | 1.435616438 |
| TCGA-EJ-A46F | 1 | 0.589041096 | 1 | 0.589041096 |
| TCGA-EJ-A46G | 0 | 1.830136986 | 0 | 1.830136986 |
| TCGA-EJ-A46H | 0 | 1.983561644 | 0 | 1.983561644 |
| TCGA-EJ-A46I | 0 | 1.830136986 | 0 | 1.830136986 |
| TCGA-EJ-A65B | 0 | 1.945205479 | 0 | 1.945205479 |
| TCGA-EJ-A65D | 0 | 1.076712329 | 0 | 1.076712329 |
| TCGA-EJ-A65E | 0 | 1.142465753 | 0 | 1.142465753 |
| TCGA-EJ-A65F | 1 | 0.205479452 | 1 | 0.205479452 |
| TCGA-EJ-A65G | 0 | 1.824657534 | 0 | 1.824657534 |
| TCGA-EJ-A65J | 0 | 1.235616438 | 0 | 1.235616438 |
| TCGA-EJ-A65M | 0 | 0.630136986 | 0 | 0.630136986 |
| TCGA-EJ-A6RA | 1 | 0.967123288 | 1 | 0.967123288 |
| TCGA-EJ-A6RC | 0 | 2.391780822 | 0 | 2.391780822 |
| TCGA-EJ-A7NF | 0 | 0.684931507 | 0 | 0.684931507 |
| TCGA-EJ-A7NG | 0 | 1.183561644 | 0 | 1.183561644 |
| TCGA-EJ-A7NH | 0 | 1.298630137 | 0 | 1.298630137 |
| TCGA-EJ-A7NJ | 0 | 0.539726027 | 0 | 0.539726027 |
| TCGA-EJ-A7NK | 0 | 1.304109589 | 0 | 1.304109589 |
| TCGA-EJ-A7NM | 0 | 0.350684932 | 0 | 0.350684932 |
| TCGA-EJ-A7NN | 1 | 0.539726027 | 1 | 0.539726027 |
| TCGA-EJ-A8FN | 0 | 0.901369863 | 0 | 0.901369863 |
| TCGA-EJ-A8FO | 0 | 0.77260274 | 0 | 0.77260274 |
| TCGA-EJ-A8FP | 1 | 0.320547945 | 1 | 0.320547945 |
| TCGA-EJ-A8FS | 1 | 0.591780822 | 1 | 0.591780822 |
| TCGA-EJ-A8FU | 0 | 0.378082192 | 0 | 0.378082192 |
| TCGA-EJ-AB20 | 0 | 0.35890411 | 0 | 0.35890411 |
| TCGA-EJ-AB27 | 0 | 0.397260274 | 0 | 0.397260274 |

| | | | | |
|---|---|---|---|---|
| TCGA-FC-7708 | 0 | 2.367123288 | 0 | 2.367123288 |
| TCGA-FC-7961 | 0 | 1.284931507 | 0 | 1.284931507 |
| TCGA-FC-A4JI | 0 | 2.402739726 | 0 | 2.402739726 |
| TCGA-FC-A5OB | 0 | 1.863013699 | 0 | 1.863013699 |
| TCGA-FC-A66V | 0 | 1.435616438 | 0 | 1.435616438 |
| TCGA-FC-A6HD | 0 | 2.161643836 | 0 | 2.161643836 |
| TCGA-FC-A8O0 | 0 | 1.687671233 | 0 | 1.687671233 |
| TCGA-G9-6329 | 0 | 3.468493151 | 0 | 3.468493151 |
| TCGA-G9-6332 | 0 | 7.279452055 | 1 | 3.232876712 |
| TCGA-G9-6333 | 0 | 6.753424658 | 0 | 6.753424658 |
| TCGA-G9-6336 | 0 | 5.665753425 | 0 | 5.665753425 |
| TCGA-G9-6338 | 0 | 5.556164384 | 0 | 5.556164384 |
| TCGA-G9-6339 | 0 | 6.989041096 | 1 | 4.476712329 |
| TCGA-G9-6342 | 0 | 4.646575342 | 0 | 4.646575342 |
| TCGA-G9-6343 | 0 | 6.712328767 | 0 | 6.712328767 |
| TCGA-G9-6347 | 0 | 5.723287671 | 0 | 5.723287671 |
| TCGA-G9-6348 | 0 | 4.150684932 | 0 | 4.150684932 |
| TCGA-G9-6351 | 0 | 5.610958904 | 0 | 5.610958904 |
| TCGA-G9-6353 | 0 | 4.224657534 | 0 | 4.224657534 |
| TCGA-G9-6354 | 0 | 5.660273973 | 0 | 5.660273973 |
| TCGA-G9-6356 | 0 | 3.928767123 | 0 | 3.928767123 |
| TCGA-G9-6361 | 0 | 3.876712329 | 0 | 3.876712329 |
| TCGA-G9-6362 | 0 | 3.953424658 | 0 | 3.953424658 |
| TCGA-G9-6363 | 0 | 3.775342466 | 0 | 3.775342466 |
| TCGA-G9-6364 | 0 | 3.282191781 | 0 | 3.282191781 |
| TCGA-G9-6365 | 0 | 3.734246575 | 0 | 3.734246575 |
| TCGA-G9-6366 | 0 | 5.334246575 | 0 | 5.334246575 |
| TCGA-G9-6367 | 0 | 3.347945205 | 0 | 3.347945205 |
| TCGA-G9-6369 | 0 | 3.328767123 | 0 | 3.328767123 |
| TCGA-G9-6370 | 0 | 3.167123288 | 0 | 3.167123288 |
| TCGA-G9-6371 | 0 | 3.35890411 | 0 | 3.35890411 |
| TCGA-G9-6373 | 0 | 2.221917808 | 0 | 2.221917808 |
| TCGA-G9-6377 | 0 | 2.624657534 | 0 | 2.624657534 |
| TCGA-G9-6378 | 0 | 3.164383562 | 0 | 3.164383562 |
| TCGA-G9-6379 | 0 | 4.871232877 | 0 | 4.871232877 |
| TCGA-G9-6384 | 0 | 2.095890411 | 0 | 2.095890411 |
| TCGA-G9-6385 | 0 | 2.273972603 | 0 | 2.273972603 |
| TCGA-G9-6494 | 0 | 4.852054795 | 0 | 4.852054795 |
| TCGA-G9-6496 | 0 | 4.728767123 | 0 | 4.728767123 |
| TCGA-G9-6498 | 0 | 5.347945205 | 1 | 3.676712329 |
| TCGA-G9-6499 | 0 | 4.22739726 | 0 | 4.22739726 |
| TCGA-G9-7509 | 0 | 4.410958904 | 0 | 4.410958904 |
| TCGA-G9-7510 | 0 | 3.246575342 | 0 | 3.246575342 |
| TCGA-G9-7519 | 0 | 2.326027397 | 0 | 2.326027397 |
| TCGA-G9-7521 | 0 | 2.580821918 | 0 | 2.580821918 |
| TCGA-G9-7522 | 0 | 2.953424658 | 0 | 2.953424658 |

| | | | | |
|---|---|---|---|---|
| TCGA-G9-7523 | 0 | 2.347945205 | 0 | 2.347945205 |
| TCGA-G9-7525 | 0 | 2.723287671 | 0 | 2.723287671 |
| TCGA-G9-A9S0 | 1 | 1.175342466 | 1 | 1.15890411 |
| TCGA-G9-A9S4 | 0 | 2.482191781 | 0 | 2.482191781 |
| TCGA-G9-A9S7 | 0 | 1.994520548 | 0 | 1.994520548 |
| TCGA-H9-7775 | 0 | 0.506849315 | 0 | 0.506849315 |
| TCGA-H9-A6BX | 0 | 2.578082192 | 0 | 2.578082192 |
| TCGA-H9-A6BY | 0 | 0.306849315 | 0 | 0.306849315 |
| TCGA-HC-7075 | 0 | 1.646575342 | 0 | 1.646575342 |
| TCGA-HC-7077 | 0 | 5.794520548 | 0 | 5.794520548 |
| TCGA-HC-7078 | 0 | 5.438356164 | 0 | 5.438356164 |
| TCGA-HC-7079 | 0 | 3.243835616 | 1 | 1.04109589 |
| TCGA-HC-7080 | 0 | 3.030136986 | 1 | 2.449315068 |
| TCGA-HC-7081 | 0 | 3.112328767 | 0 | 3.112328767 |
| TCGA-HC-7209 | 0 | 1.205479452 | 0 | 1.205479452 |
| TCGA-HC-7210 | 0 | 2.378082192 | 0 | 2.378082192 |
| TCGA-HC-7211 | 0 | 3.345205479 | 0 | 3.345205479 |
| TCGA-HC-7212 | 0 | 2.383561644 | 0 | 2.383561644 |
| TCGA-HC-7213 | 1 | 0.465753425 | 1 | 0.465753425 |
| TCGA-HC-7230 | 0 | 2.742465753 | 0 | 2.742465753 |
| TCGA-HC-7231 | 0 | 2.57260274 | 0 | 2.57260274 |
| TCGA-HC-7232 | 0 | 3.180821918 | 1 | 2.098630137 |
| TCGA-HC-7233 | 0 | 3.712328767 | 0 | 3.712328767 |
| TCGA-HC-7736 | 0 | 3.021917808 | 0 | 3.021917808 |
| TCGA-HC-7737 | 0 | 2.98630137 | 0 | 2.98630137 |
| TCGA-HC-7738 | 0 | 2.64109589 | 1 | 1.150684932 |
| TCGA-HC-7740 | 0 | 1.964383562 | 0 | 1.964383562 |
| TCGA-HC-7742 | 0 | 2.569863014 | 0 | 2.569863014 |
| TCGA-HC-7744 | 0 | 1.454794521 | 0 | 1.454794521 |
| TCGA-HC-7745 | 0 | 1.975342466 | 0 | 1.975342466 |
| TCGA-HC-7747 | 0 | 2.049315068 | 0 | 2.049315068 |
| TCGA-HC-7748 | 0 | 2.169863014 | 0 | 2.169863014 |
| TCGA-HC-7749 | 0 | 1.983561644 | 0 | 1.983561644 |
| TCGA-HC-7750 | 0 | 1.430136986 | 0 | 1.430136986 |
| TCGA-HC-7752 | 0 | 2.142465753 | 0 | 2.142465753 |
| TCGA-HC-7817 | 0 | 2.542465753 | 0 | 2.542465753 |
| TCGA-HC-7818 | 0 | 2.569863014 | 0 | 2.569863014 |
| TCGA-HC-7819 | 0 | 1.487671233 | 0 | 1.487671233 |
| TCGA-HC-7820 | 0 | 2.046575342 | 0 | 2.046575342 |
| TCGA-HC-7821 | 0 | 2.619178082 | 0 | 2.619178082 |
| TCGA-HC-8213 | 0 | 1.61369863 | 0 | 1.61369863 |
| TCGA-HC-8216 | 0 | 1.868493151 | 0 | 1.868493151 |
| TCGA-HC-8256 | 0 | 2.356164384 | 0 | 2.356164384 |
| TCGA-HC-8257 | 0 | 1.989041096 | 0 | 1.989041096 |
| TCGA-HC-8258 | 0 | 2.320547945 | 0 | 2.320547945 |

| | | | | |
|---|---|---|---|---|
| TCGA-HC-8259 | 0 | 2.531506849 | 0 | 2.531506849 |
| TCGA-HC-8260 | 0 | 1.879452055 | 0 | 1.879452055 |
| TCGA-HC-8261 | 0 | 1.495890411 | 0 | 1.495890411 |
| TCGA-HC-8262 | 0 | 1.860273973 | 0 | 1.860273973 |
| TCGA-HC-8264 | 0 | 0.131506849 | 0 | 0.131506849 |
| TCGA-HC-8265 | 0 | 1.323287671 | 0 | 1.323287671 |
| TCGA-HC-8266 | 0 | 1.295890411 | 0 | 1.295890411 |
| TCGA-HC-A48F | 0 | 1.863013699 | 0 | 1.863013699 |
| TCGA-HC-A631 | 0 | 0.147945205 | 0 | 0.147945205 |
| TCGA-HC-A632 | 0 | 0.167123288 | 0 | 0.167123288 |
| TCGA-HC-A6AL | 0 | 0.183561644 | 0 | 0.183561644 |
| TCGA-HC-A6AN | 0 | 0.134246575 | 0 | 0.134246575 |
| TCGA-HC-A6AO | 0 | 1.452054795 | 0 | 1.452054795 |
| TCGA-HC-A6AP | 0 | 0.194520548 | 0 | 0.194520548 |
| TCGA-HC-A6AQ | 0 | 0.290410959 | 0 | 0.290410959 |
| TCGA-HC-A6AS | 0 | 0.120547945 | 0 | 0.120547945 |
| TCGA-HC-A6HX | 0 | 0.104109589 | 0 | 0.104109589 |
| TCGA-HC-A6HY | 0 | 0.367123288 | 0 | 0.367123288 |
| TCGA-HC-A76W | 0 | 0.583561644 | 0 | 0.583561644 |
| TCGA-HC-A76X | 0 | 0.410958904 | 0 | 0.410958904 |
| TCGA-HC-A8CY | 0 | 0.794520548 | 0 | 0.794520548 |
| TCGA-HC-A8D0 | 0 | 2.123287671 | 0 | 2.123287671 |
| TCGA-HC-A8D1 | 0 | 1.569863014 | 0 | 1.569863014 |
| TCGA-HC-A9TE | 0 | 1.608219178 | 1 | 0.591780822 |
| TCGA-HC-A9TH | 0 | 2.312328767 | 1 | 0.961643836 |
| TCGA-HI-7168 | 1 | 0.843835616 | 1 | 6.863013699 |
| TCGA-HI-7169 | 0 | 7.353424658 | 0 | 7.353424658 |
| TCGA-HI-7170 | 0 | 6.909589041 | 0 | 6.909589041 |
| TCGA-HI-7171 | 1 | 0.597260274 | 1 | 3.64109589 |
| TCGA-J4-8198 | 0 | 1.682191781 | 0 | 1.682191781 |
| TCGA-J4-8200 | 0 | 3.336986301 | 0 | 3.336986301 |
| TCGA-J4-A67K | 0 | 2.750684932 | 0 | 2.750684932 |
| TCGA-J4-A67L | 0 | 2.109589041 | 0 | 2.109589041 |
| TCGA-J4-A67M | 0 | 2.578082192 | 0 | 2.578082192 |
| TCGA-J4-A67N | 1 | 1.210958904 | 1 | 1.210958904 |
| TCGA-J4-A67O | 0 | 2.298630137 | 0 | 2.298630137 |
| TCGA-J4-A67Q | 0 | 2.671232877 | 0 | 2.671232877 |
| TCGA-J4-A67R | 0 | 2.493150685 | 0 | 2.493150685 |
| TCGA-J4-A67S | 0 | 2.282191781 | 1 | 1.939726027 |
| TCGA-J4-A67T | 0 | 0.501369863 | 0 | 0.501369863 |
| TCGA-J4-A6G1 | 0 | 2.106849315 | 0 | 2.106849315 |
| TCGA-J4-A6G3 | 0 | 2.326027397 | 1 | 1.693150685 |
| TCGA-J4-A6M7 | 0 | 1.402739726 | 0 | 1.402739726 |
| TCGA-J4-A83I | 0 | 1.876712329 | 0 | 1.876712329 |
| TCGA-J4-A83J | 0 | 1.906849315 | 0 | 1.906849315 |
| TCGA-J4-A83K | 0 | 0.898630137 | 0 | 0.898630137 |

| | | | | |
|---|---|---|---|---|
| TCGA-J4-A83L | 0 | 1.97260274 | 0 | 1.97260274 |
| TCGA-J4-A83M | 0 | 1.487671233 | 1 | 1.42739726 |
| TCGA-J4-A83N | 0 | 2.717808219 | 1 | 1.18630137 |
| TCGA-J4-AATV | 0 | 1.515068493 | 0 | 1.515068493 |
| TCGA-J4-AATZ | 0 | 1.128767123 | 1 | 0.216438356 |
| TCGA-J4-AAU2 | 0 | 2.260273973 | 0 | 2.260273973 |
| TCGA-J9-A52B | 0 | 1.156164384 | 1 | 0.156164384 |
| TCGA-J9-A52C | 0 | 0.487671233 | 0 | 0.487671233 |
| TCGA-J9-A52D | 0 | 0.580821918 | 0 | 0.580821918 |
| TCGA-J9-A52E | 0 | 0.884931507 | 0 | 0.884931507 |
| TCGA-J9-A8CK | 0 | 0.879452055 | 0 | 0.879452055 |
| TCGA-J9-A8CL | 1 | 0.361643836 | 1 | 0.361643836 |
| TCGA-J9-A8CM | 1 | 0.942465753 | 1 | 0.942465753 |
| TCGA-J9-A8CN | 0 | 3.389041096 | 0 | 3.389041096 |
| TCGA-J9-A8CP | 0 | 1.057534247 | 0 | 1.057534247 |
| TCGA-KC-A4BL | 1 | 0.528767123 | 1 | 0.528767123 |
| TCGA-KC-A4BN | 0 | 4.97260274 | 0 | 4.97260274 |
| TCGA-KC-A4BR | 0 | 3.695890411 | 1 | 2.8 |
| TCGA-KC-A4BV | 1 | 3.638356164 | 1 | 3.638356164 |
| TCGA-KC-A7F3 | 0 | 1.821917808 | 0 | 1.821917808 |
| TCGA-KC-A7F5 | 0 | 0.249315068 | 0 | 0.249315068 |
| TCGA-KC-A7F6 | 0 | 0.720547945 | 0 | 0.720547945 |
| TCGA-KC-A7FA | 0 | 1.638356164 | 0 | 1.638356164 |
| TCGA-KC-A7FD | 0 | 0.739726027 | 0 | 0.739726027 |
| TCGA-KC-A7FE | 0 | 0.964383562 | 0 | 0.964383562 |
| TCGA-KK-A59V | 0 | 9.424657534 | 0 | 9.424657534 |
| TCGA-KK-A59X | 0 | 6.945205479 | 1 | 6.117808219 |
| TCGA-KK-A59Y | 0 | 5.391780822 | 0 | 5.391780822 |
| TCGA-KK-A59Z | 0 | 7.2 | 0 | 7.2 |
| TCGA-KK-A5A1 | 1 | 6.764383562 | 1 | 0.556164384 |
| TCGA-KK-A6DY | 0 | 11.76712329 | 0 | 11.76712329 |
| TCGA-KK-A6E0 | 1 | 2.578082192 | 1 | 2.578082192 |
| TCGA-KK-A6E1 | 0 | 6.021917808 | 0 | 6.021917808 |
| TCGA-KK-A6E2 | 0 | 13.76438356 | 0 | 13.76438356 |
| TCGA-KK-A6E3 | 0 | 5.632876712 | 0 | 5.632876712 |
| TCGA-KK-A6E4 | 1 | 9.594520548 | 0 | 9.594520548 |
| TCGA-KK-A6E5 | 0 | 5.679452055 | 0 | 5.679452055 |
| TCGA-KK-A6E6 | 0 | 9.443835616 | 0 | 9.443835616 |
| TCGA-KK-A6E7 | 0 | 7.561643836 | 1 | 2.534246575 |
| TCGA-KK-A6E8 | 0 | 4.849315068 | 0 | 4.849315068 |
| TCGA-KK-A7AP | 0 | 0.536986301 | 0 | 0.536986301 |
| TCGA-KK-A7AQ | 0 | 4.410958904 | 1 | 3.334246575 |
| TCGA-KK-A7AU | 0 | 4.863013699 | 1 | 0.567123288 |
| TCGA-KK-A7AV | 0 | 2.252054795 | 0 | 2.252054795 |
| TCGA-KK-A7AW | 1 | 0.55890411 | 0 | 2.887671233 |

| | | | | |
|---|---|---|---|---|
| TCGA-KK-A7AY | 0 | 4.805479452 | 1 | 3.079452055 |
| TCGA-KK-A7AZ | 0 | 4.161643836 | 0 | 4.161643836 |
| TCGA-KK-A7B0 | 0 | 3.64109589 | 1 | 1.660273973 |
| TCGA-KK-A7B1 | 0 | 2.912328767 | 0 | 2.912328767 |
| TCGA-KK-A7B2 | 0 | 3.010958904 | 1 | 1.895890411 |
| TCGA-KK-A7B3 | 0 | 2.463013699 | 1 | 0.805479452 |
| TCGA-KK-A7B4 | 1 | 1.745205479 | 1 | 1.745205479 |
| TCGA-KK-A8I4 | 0 | 6.189041096 | 1 | 3.183561644 |
| TCGA-KK-A8I5 | 0 | 8.482191781 | 0 | 8.482191781 |
| TCGA-KK-A8I6 | 0 | 1.830136986 | 0 | 1.830136986 |
| TCGA-KK-A8I7 | 0 | 5.484931507 | 1 | 2.980821918 |
| TCGA-KK-A8I8 | 0 | 2.649315068 | 0 | 2.649315068 |
| TCGA-KK-A8I9 | 0 | 2.742465753 | 1 | 2.575342466 |
| TCGA-KK-A8IA | 0 | 5.290410959 | 0 | 5.290410959 |
| TCGA-KK-A8IB | 0 | 0.22739726 | 0 | 0.22739726 |
| TCGA-KK-A8IC | 1 | 2.904109589 | 1 | 2.904109589 |
| TCGA-KK-A8ID | 0 | 5.77260274 | 0 | 5.77260274 |
| TCGA-KK-A8IF | 1 | 1.775342466 | 1 | 1.775342466 |
| TCGA-KK-A8IG | 0 | 6.863013699 | 0 | 6.863013699 |
| TCGA-KK-A8IH | 0 | 5.515068493 | 0 | 5.515068493 |
| TCGA-KK-A8II | 1 | 1.715068493 | 1 | 1.715068493 |
| TCGA-KK-A8IJ | 0 | 4.271232877 | 1 | 0.84109589 |
| TCGA-KK-A8IK | 0 | 5.621917808 | 0 | 5.621917808 |
| TCGA-KK-A8IL | 1 | 1.720547945 | 0 | 1.720547945 |
| TCGA-KK-A8IM | 0 | 5.246575342 | 0 | 5.246575342 |
| TCGA-M7-A71Y | 0 | 1.350684932 | 0 | 1.350684932 |
| TCGA-M7-A71Z | 0 | 1.761643836 | 0 | 1.761643836 |
| TCGA-M7-A720 | 0 | 1.052054795 | 0 | 1.052054795 |
| TCGA-M7-A721 | 0 | 1.095890411 | 0 | 1.095890411 |
| TCGA-M7-A722 | 1 | 1.531506849 | 1 | 1.531506849 |
| TCGA-M7-A723 | 0 | 2.090410959 | 0 | 2.090410959 |
| TCGA-M7-A724 | 0 | 2.435616438 | 0 | 2.435616438 |
| TCGA-M7-A725 | 0 | 1.569863014 | 0 | 1.569863014 |
| TCGA-MG-AAMC | 0 | 0.473972603 | 0 | 0.473972603 |
| TCGA-QU-A6IL | 0 | 0.265753425 | 0 | 0.265753425 |
| TCGA-QU-A6IM | 0 | 3.416438356 | 0 | 3.416438356 |
| TCGA-QU-A6IN | 0 | 11.68219178 | 0 | 11.68219178 |
| TCGA-QU-A6IO | 0 | 10.18082192 | 0 | 10.18082192 |
| TCGA-QU-A6IP | 0 | 7.178082192 | 0 | 7.178082192 |
| TCGA-SU-A7E7 | 0 | 1.509589041 | 0 | 1.509589041 |
| TCGA-TP-A8TT | 0 | 1.44109589 | 0 | 1.44109589 |
| TCGA-TP-A8TV | 0 | 1.671232877 | 0 | 1.671232877 |
| TCGA-V1-A8MF | 0 | 3.969863014 | 0 | 3.969863014 |
| TCGA-V1-A8MG | 0 | 2.347945205 | 0 | 2.347945205 |
| TCGA-V1-A8ML | 0 | 1.22739726 | 0 | 1.22739726 |
| TCGA-V1-A8MM | 1 | 2.712328767 | 1 | 2.712328767 |

| | | | | |
|---|---|---|---|---|
| TCGA-V1-A8MU | 0 | 5.139726027 | 0 | 5.139726027 |
| TCGA-V1-A8WL | 0 | 5.336986301 | 0 | 5.336986301 |
| TCGA-V1-A8WN | 0 | 2.531506849 | 0 | 2.531506849 |
| TCGA-V1-A8WS | 0 | 1.298630137 | 0 | 1.298630137 |
| TCGA-V1-A8WV | 0 | 1.879452055 | 0 | 1.879452055 |
| TCGA-V1-A8WW | 0 | 2.953424658 | 0 | 2.953424658 |
| TCGA-V1-A8X3 | 0 | 0.553424658 | 0 | 0.553424658 |
| TCGA-V1-A905 | 1 | 0.339726027 | 1 | 0.339726027 |
| TCGA-V1-A907 | 1 | 2.523287671 | 1 | 2.243835616 |
| TCGA-V1-A90A | 0 | 1.742465753 | 0 | 1.742465753 |
| TCGA-V1-A90F | 0 | 3.243835616 | 0 | 3.243835616 |
| TCGA-V1-A90H | 0 | 6.583561644 | 0 | 6.583561644 |
| TCGA-V1-A90L | 0 | 5.24109589 | 1 | 0.287671233 |
| TCGA-V1-A90Q | 0 | 1.016438356 | 0 | 1.016438356 |
| TCGA-V1-A90X | 0 | 3.106849315 | 0 | 3.106849315 |
| TCGA-V1-A90Y | 0 | 2.923287671 | 0 | 2.923287671 |
| TCGA-V1-A9Z8 | 0 | 2.265753425 | 0 | 2.265753425 |
| TCGA-V1-A9Z9 | 0 | 1.479452055 | 0 | 1.479452055 |
| TCGA-V1-A9ZG | 0 | 4.156164384 | 0 | 4.156164384 |
| TCGA-V1-A9ZI | 1 | 0.235616438 | 0 | 4.493150685 |
| TCGA-V1-A9ZK | 0 | 3.789041096 | 0 | 3.789041096 |
| TCGA-VN-A88I | 0 | 0.736986301 | 0 | 0.736986301 |
| TCGA-VN-A88K | 0 | 2.126027397 | 0 | 2.126027397 |
| TCGA-VN-A88L | 0 | 2.043835616 | 0 | 2.043835616 |
| TCGA-VN-A88M | 0 | 0.517808219 | 0 | 0.517808219 |
| TCGA-VN-A88N | 0 | 2.098630137 | 0 | 2.098630137 |
| TCGA-VN-A88O | 0 | 1.317808219 | 0 | 1.317808219 |
| TCGA-VN-A88P | 0 | 3.452054795 | 0 | 3.452054795 |
| TCGA-VN-A88Q | 0 | 3.742465753 | 0 | 3.742465753 |
| TCGA-VN-A88R | 0 | 2.147945205 | 1 | 1.402739726 |
| TCGA-VN-A943 | 0 | 1.35890411 | 0 | 1.35890411 |
| TCGA-VP-A872 | 0 | 9.947945205 | 0 | 9.947945205 |
| TCGA-VP-A875 | 0 | 6.326027397 | 0 | 6.326027397 |
| TCGA-VP-A876 | 0 | 9.131506849 | 0 | 9.131506849 |
| TCGA-VP-A878 | 0 | 8.575342466 | 1 | 0.268493151 |
| TCGA-VP-A879 | 1 | 1.994520548 | 0 | 1.994520548 |
| TCGA-VP-A87B | 0 | 7.457534247 | 1 | 6.775342466 |
| TCGA-VP-A87C | 0 | 4.630136986 | 0 | 4.630136986 |
| TCGA-VP-A87D | 1 | 3.271232877 | 1 | 3.271232877 |
| TCGA-VP-A87E | 0 | 5.580821918 | 0 | 5.580821918 |
| TCGA-VP-A87H | 0 | 1.816438356 | 0 | 1.816438356 |
| TCGA-VP-A87J | 0 | 4.860273973 | 0 | 4.860273973 |
| TCGA-VP-A87K | 1 | 1.295890411 | 1 | 1.460273973 |
| TCGA-VP-AA1N | 0 | 3.739726027 | 0 | 3.739726027 |
| TCGA-WW-A8ZI | 0 | 1.230136986 | 0 | 1.230136986 |

| | | | | |
|---|---|---|---|---|
| TCGA-X4-A8KQ | 0 | 3.789041096 | 0 | 3.789041096 |
| TCGA-X4-A8KS | 0 | 2.682191781 | 0 | 2.682191781 |
| TCGA-XA-A8JR | 0 | 1.030136986 | 0 | 1.030136986 |
| TCGA-XJ-A83F | 0 | 3.087671233 | 0 | 3.087671233 |
| TCGA-XJ-A83G | 0 | 4.002739726 | 0 | 4.002739726 |
| TCGA-XJ-A83H | 0 | 3.506849315 | 0 | 3.506849315 |
| TCGA-XJ-A9DI | 0 | 4.002739726 | 0 | 4.002739726 |
| TCGA-XJ-A9DK | 0 | 0.753424658 | 0 | 0.753424658 |
| TCGA-XJ-A9DQ | 0 | 0.249315068 | 0 | 0.249315068 |
| TCGA-XJ-A9DX | 0 | 2.665753425 | 0 | 2.665753425 |
| TCGA-XK-AAIR | 0 | 2.663013699 | 0 | 2.663013699 |
| TCGA-XK-AAIV | 0 | 3.104109589 | 0 | 3.104109589 |
| TCGA-XK-AAIW | 0 | 3.336986301 | 1 | 1.169863014 |
| TCGA-XK-AAJA | 0 | 2.547945205 | 0 | 2.547945205 |
| TCGA-XK-AAJP | 0 | 3.449315068 | 0 | 3.449315068 |
| TCGA-XK-AAJR | 1 | 0.35890411 | 1 | 0.35890411 |
| TCGA-XK-AAJT | 0 | 3.928767123 | 0 | 3.928767123 |
| TCGA-XK-AAJU | 0 | 4.317808219 | 0 | 4.317808219 |
| TCGA-XQ-A8TB | 0 | 2.109589041 | 0 | 2.109589041 |
| TCGA-Y6-A8TL | 0 | 2.717808219 | 0 | 2.717808219 |
| TCGA-Y6-A9XI | 0 | 1.44109589 | 0 | 1.44109589 |
| TCGA-YJ-A8SW | 1 | 0.405479452 | 0 | 0.405479452 |
| TCGA-YL-A8HJ | 0 | 4.41369863 | 0 | 4.41369863 |
| TCGA-YL-A8HK | 1 | 3.769863014 | 1 | 3.769863014 |
| TCGA-YL-A8HL | 0 | 4.287671233 | 0 | 4.287671233 |
| TCGA-YL-A8HM | 0 | 4.060273973 | 1 | 4.035616438 |
| TCGA-YL-A8HO | 0 | 6.312328767 | 1 | 2.926027397 |
| TCGA-YL-A8S8 | 1 | 1.860273973 | 1 | 1.860273973 |
| TCGA-YL-A8S9 | 0 | 5.046575342 | 1 | 2.756164384 |
| TCGA-YL-A8SA | 0 | 1.402739726 | 0 | 1.402739726 |
| TCGA-YL-A8SB | 0 | 4.709589041 | 1 | 3.791780822 |
| TCGA-YL-A8SC | 0 | 3.454794521 | 1 | 0.416438356 |
| TCGA-YL-A8SH | 1 | 2.397260274 | 0 | 2.397260274 |
| TCGA-YL-A8SI | 0 | 4.421917808 | 1 | 3.898630137 |
| TCGA-YL-A8SJ | 1 | 2.060273973 | 1 | 2.060273973 |
| TCGA-YL-A8SK | 0 | 3.857534247 | 0 | 3.857534247 |
| TCGA-YL-A8SL | 0 | 2.704109589 | 0 | 2.704109589 |
| TCGA-YL-A8SO | 0 | 9.531506849 | 0 | 9.531506849 |
| TCGA-YL-A8SP | 0 | 6.482191781 | 1 | 5.578082192 |
| TCGA-YL-A8SQ | 1 | 0.901369863 | 1 | 0.901369863 |
| TCGA-YL-A8SR | 0 | 0.747945205 | 0 | 0.747945205 |
| TCGA-YL-A9WH | 0 | 1.169863014 | 0 | 1.169863014 |
| TCGA-YL-A9WI | 0 | 4.446575342 | 0 | 4.446575342 |
| TCGA-YL-A9WJ | 1 | 0.208219178 | 1 | 4.043835616 |
| TCGA-YL-A9WK | 1 | 2.764383562 | 1 | 2.764383562 |
| TCGA-YL-A9WL | 1 | 2.02739726 | 1 | 2.02739726 |

| | | | | |
|---|---|---|---|---|
| TCGA-YL-A9WX | 1 | 4.126027397 | 1 | 4.126027397 |
| TCGA-YL-A9WY | 1 | 2.095890411 | 1 | 2.095890411 |
| TCGA-ZG-A8QW | 0 | 0.257534247 | 0 | 0.257534247 |
| TCGA-ZG-A8QX | 0 | 1.210958904 | 0 | 1.210958904 |
| TCGA-ZG-A8QY | 0 | 1.106849315 | 0 | 1.106849315 |
| TCGA-ZG-A8QZ | 0 | 0.843835616 | 0 | 0.843835616 |
| TCGA-ZG-A9KY | 0 | 0.356164384 | 0 | 0.356164384 |
| TCGA-ZG-A9L0 | 0 | 0.97260274 | 0 | 0.97260274 |
| TCGA-ZG-A9L1 | 0 | 3.567123288 | 0 | 3.567123288 |
| TCGA-ZG-A9L2 | 1 | 0.493150685 | 1 | 0.493150685 |
| TCGA-ZG-A9L4 | 0 | 3.035616438 | 0 | 3.035616438 |
| TCGA-ZG-A9L5 | 0 | 2.487671233 | 0 | 2.487671233 |
| TCGA-ZG-A9L6 | 1 | 1.819178082 | 1 | 1.819178082 |
| TCGA-ZG-A9L9 | 0 | 1.690410959 | 1 | 0.139726027 |
| TCGA-ZG-A9LB | 0 | 1.597260274 | 0 | 1.597260274 |
| TCGA-ZG-A9LM | 0 | 1.035616438 | 0 | 1.035616438 |
| TCGA-ZG-A9LN | 0 | 0.315068493 | 0 | 0.315068493 |
| TCGA-ZG-A9LS | 0 | 1.41369863 | 0 | 1.41369863 |
| TCGA-ZG-A9LU | 0 | 1.58630137 | 0 | 1.58630137 |
| TCGA-ZG-A9LY | 0 | 1.01369863 | 0 | 1.01369863 |
| TCGA-ZG-A9LZ | 1 | 0.849315068 | 0 | 1.895890411 |
| TCGA-ZG-A9M4 | 0 | 1.498630137 | 0 | 1.498630137 |
| TCGA-ZG-A9MC | 0 | 1.246575342 | 0 | 1.246575342 |
| TCGA-ZG-A9N3 | 0 | 0.956164384 | 0 | 0.956164384 |
| TCGA-ZG-A9ND | 0 | 1.123287671 | 0 | 1.123287671 |
| TCGA-ZG-A9NI | 0 | 0.361643836 | 0 | 0.361643836 |
| TCGA-EJ-7312 | 0 | 3.010958904 | NA | NA |
| TCGA-XK-AAJ3 | 0 | 3.457534247 | NA | NA |
| TCGA-XK-AAK1 | 0 | 2.682191781 | NA | NA |

DFS: disease-free survival event, 1 for patient having new tumor event whether it is a local recurrence, distant metastasis, new primary tumor of the cancer, including cases with a new tumor event whose type is N/A. Disease free was defined by: first, treatment_outcome_first_course is "Complete Remission/Response"; if the tumor type doesn't have "treatment_outcome_first_course" then disease-free was defined by the value "R0" in the field of "residual_tumor"; otherwise, disease-free was defined by the value "negative" in the field of "margin_status". If the tumor type did not have any of these fields, then its DFI was NA.
DFS.time: disease-free survival time in days, new_tumor_event_dx_days_to for events, or for censored cases, either last_contact_days_to or death_days_to, whichever is applicable.
PFI: progression-free interval event, 1 for patient having new tumor event whether it was a progression of disease, local recurrence, distant metastasis, new primary tumors all sites , or died with the cancer without new tumor event, including cases with a new tumor event whose

type is N/A.
PFI.time: progression-free interval time in days, for events, either new_tumor_event_dx_days_to or death_days_to, whichever is applicable; or for censored cases, either last_contact_days_to or death_days_to, whichever is applicable.
